# Topographic distribution of inflammation factors in a healing aneurysm

**DOI:** 10.1186/s12974-023-02863-1

**Published:** 2023-08-02

**Authors:** Basil E. Grüter, Gwendoline Canzanella, Joshua Hägler, Jeannine Rey, Stefan Wanderer, Michael von Gunten, José A. Galvan, Rainer Grobholz, Hans-Rudolf Widmer, Luca Remonda, Lukas Andereggen, Serge Marbacher

**Affiliations:** 1 Division of Neuroradiology, Department of Radiology, Kantonsspital Aarau, C/o NeuroResearch Office,Tellstrasse 1, 5001 Aarau, Switzerland; 2 Department of Neurosurgery, Kantonsspital Aarau, Aarau, Switzerland; 3 Program for Regenerative Neuroscience, Department for BioMedical Research, University of Bern, Bern, Switzerland; 4 Institute of Pathology Laenggasse, Ittigen, Switzerland; 5 Translational Research Unit (TRU), Institute of Pathology, University of Bern, Bern, Switzerland; 6 Institute of Pathology, Kantonsspital Aarau, Aarau, Switzerland; 7 Medical Faculty, University of Zurich, Zurich, Switzerland

**Keywords:** Endovascular procedures, Inflammation, Intracranial aneurysm, Models, Animal, Neointima

## Abstract

**Background:**

Healing of intracranial aneurysms following endovascular treatment relies on the organization of early thrombus into mature scar tissue and neointima formation. Activation and deactivation of the inflammation cascade plays an important role in this process. In addition to timely evolution, its topographic distribution is hypothesized to be crucial for successful aneurysm healing.

**Methods:**

Decellularized saccular sidewall aneurysms were created in Lewis rats and coiled. At follow-up (after 3 days (*n* = 16); 7 days (*n* = 19); 21 days (*n* = 8)), aneurysms were harvested and assessed for healing status. In situ hybridization was performed for soluble inflammatory markers (IL6, MMP2, MMP9, TNF-α, FGF23, VEGF), and immunohistochemical analysis to visualize inflammatory cells (CD45, CD3, CD20, CD31, CD163, HLA-DR). These markers were specifically documented for five regions of interest: aneurysm neck, dome, neointima, thrombus, and adjacent vessel wall.

**Results:**

Coiled aneurysms showed enhanced patterns of thrombus organization and neointima formation, whereas those without treatment demonstrated heterogeneous patterns of thrombosis, thrombus recanalization, and aneurysm growth (*p* = 0.02). In coiled aneurysms, inflammation markers tended to accumulate inside the thrombus and in the neointima (*p* < 0.001). Endothelial cells accumulated directly in the neointima (*p* < 0.0001), and their presence was associated with complete aneurysm healing.

**Conclusion:**

The presence of proinflammatory cells plays a crucial role in aneurysm remodeling after coiling. Whereas thrombus organization is hallmarked by a pronounced intra-thrombotic inflammatory reaction, neointima maturation is characterized by direct invasion of endothelial cells. Knowledge concerning topographic distribution of regenerative inflammatory processes may pave the way for future treatment modalities which enhance aneurysm healing after endovascular therapy.

**Supplementary Information:**

The online version contains supplementary material available at 10.1186/s12974-023-02863-1.

## Introduction

Aneurysm healing following endovascular treatment (EVT) relies on thrombus organization and formation of an endothelialized neointima. This process is predominantly mediated by migration of cells from the myofibroblasts line, which originate in the adjacent vessel and aneurysm wall [1, 2]. Activation and deactivation of the inflammation cascade plays an important role in initiating and directing cell migration [3]. The situation is complicated as pathogenesis of intracranial aneurysms (IA) is associated with mural cell loss, which in turn triggers chronic aneurysm wall inflammation [4, 5]. Furthermore, inert and, in particular, bioactive endovascular materials (such as stents) may trigger a prolonged local inflammatory reaction which impairs biological IA healing [6, 7]. Whereas some locoregional acute inflammation is necessary to initiate the aneurysm healing process, prolonged and overriding inflammation may lead to ongoing thrombus remodeling without thrombus maturation—causing residual aneurysm perfusion or further aneurysm growth [8, 9]. Finally, the presence of proinflammatory cytokines and cell types follows a distinct temporal cascade in healing aneurysms after EVT [10].

Healing and permanent occlusion following EVT is exceptionally challenging in rupture-prone IAs with highly degenerated walls [9, 11]. In fact, both human histopathological and experimental studies have revealed inconsistent neointima formation and a heterogenous pattern of thrombus organization between the aneurysm dome and its neck in hypocellular IAs after EVT [4, 12]. In addition to timely evolution, a spatial-topographic distribution of inflammation factors is therefore thought to be crucial for successful IA healing. This study investigates the topographic distribution of inflammation and healing markers after EVT in decellularized experimental rat saccular sidewall aneurysms—specifically in the following regions of interest (ROIs): aneurysm neck, aneurysm dome, thrombus interior, neointima, and adjacent vessel wall.

## Materials and methods

### Study design and animals

**Fig. 1:**
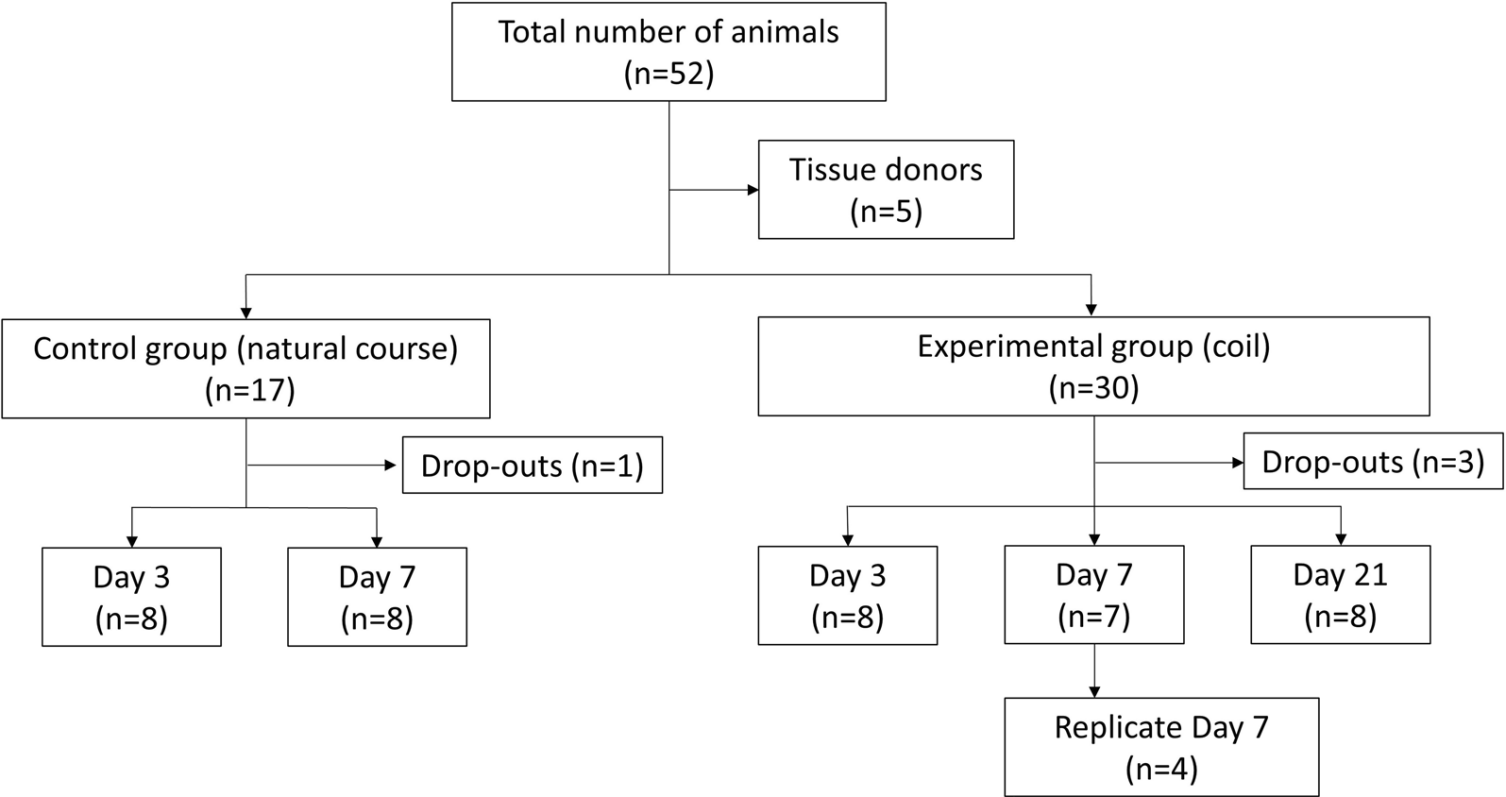
Study design flowchart. Of a total of *n* = 52 animals, *n* = 5 were used as tissue donors and *n* = 4 were excluded due to morbidity (*n* = 2, postoperative paraplegia) or premature mortality (*n* = 2: *n* = 1 anesthesia related, *n* = 1 unclear). No 21-day follow-ups were performed in the control group as the model is associated with high rates of aneurysm growth and spontaneous rupture in this timeframe [4]

A total of *n* = 52 *male Lewis* rats (Janvier labs, Le Genest-Saint-Isle, France) aged 12 weeks or older were included in this study. All animals were housed in groups of 4 in a special room at 22–24 °C and 12-h light/dark cycle with unlimited access to a pellet diet and tap water. They received humane care in accordance with institutional guidelines. After surgical creation of sidewall aneurysms, animals were randomly allocated to either an experimental (coil treatment) or control group (natural course) and followed for 3, 7 or 21 days, at which time tissue was harvested for further analysis (Fig. 1). The 21-day follow-up was omitted in the control group for ethical reasons as the model used had previously shown high rates of aneurysm growth and spontaneous rupture after 7 days [4]. With inflammation peaking 7 days after coiling, a reproduction cohort (*n* = 4) was conducted for this group. A priori sample size calculations revealed an ideal group size of *n* = 8 (Additional file 1). Experiments were approved by the local animal welfare committee (BE 60/19) and conducted in accordance with ARRIVE guidelines [13].

### Anesthesia, aneurysm formation, and treatment

Rats were placed in a gas chamber to inhale isoflurane 4% until loss of consciousness, weighed (340 ± 15 g), and injected with a mixture of fentanyl (Sintetica Switzerland) 0.005 mg/kg + medetomidine (Medetor, Virbac, UK) 0.15 mg/kg + midazolam (Dormicum, Roche, Switzerland) 2 mg/kg s.c. Vital parameters (heart rate, arterial oxygen saturation, breath rate, and temperature) were continuously monitored during surgery (MouseOx, Starr Life Sciences Corp., Oakmont, USA). Anesthesia was maintained with isoflurane 1% administered via an O_2_ mask. Overall, *n* = 5 animals were used as tissue donors and saccular sidewall aneurysms were created in *n* = 47 animals as previously described [14]. In brief, a standardized piece of donor animal thoracic aorta was ligated, chemically decellularized in sodium dodecyl sulfate (SDS), and sutured in an end-to-side constellation to the abdominal aorta of a recipient animal. Coiling was performed during aneurysm creation surgery, with the final quadrant of the anastomosis still open, using 2 cm (3 mm diameter) of a Target 360 TM Ultra coil (Stryker, Kalamazoo, MI, USA). Following surgery reversal of anesthesia was achieved via subcutaneous injection of buprenorphine (Temgesic, Indivior, Switzerland) 0.05 mg/kg + atipamezole (Revertor, Virbac, UK) 0.75 mg/kg + flumazenil (Labatec, Switzerland) 0.2 mg/kg. Postoperative analgesia was provided via administration of meloxicam (Metacam, Boehringer Ingelheim, Germany) 1.5 mg/kg (s.c.) upon return to consciousness and then twice a day for three days following surgery. Glucose 5% (B.Braun, Switzerland) and buprenorphine (Temgesic, Indivior, Switzerland) 0.3 mg/ml was added to the drinking water as a baseline adjunction for 1 week following surgery. Buprenorphine 0.05 mg/kg was given as rescue analgesia as often as needed.

### Exclusion criteria, tissue preparation and macroscopic inspection

Four animals were excluded from analysis due to premature death (*n* = 2: *n* = 1 related to anesthesia, *n* = 1 unclear) or morbidity (premature euthanasia without tissue harvesting in *n* = 2 animals with postoperative paraplegia). The remaining animals were euthanized at a pre-defined point with an overdose of intracardial ketamine hydrochloride injection (Narketan, Vetoquinol, Switzerland, 120 mg/kg). Aneurysms were harvested and measured in all dimensions, and the posterior aorta was opened to inspect the aneurysm orifice. Tissues were immediately fixed in 4% paraformaldehyde for embedding in paraffin (FFPE, J.T. Baker, Arnhem, The Netherlands).

### Light microscopy

Paraffin-embedded aneurysms were cut in 2-μm slices parallel to the underlying parent artery. For staining standard protocols used included hematoxylin–eosin (HE), Masson–Goldner trichrome (MASA), and immune staining for smooth muscle actin (SMA), and Von Willebrand factor (F VIII).

### In situ hybridization

In situ hybridization (ISH) was performed to detect mRNA, through automated staining using Bond RX (Leica Biosystems) and RNAscope® technology (Advanced Cell Diagnostics, Hayward, CA, USA). All slides were dewaxed in Bond Dewax Solution (product code AR9222, Leica Biosystems) and heat-induced epitope retrieval at pH 9 in Tris Buffer (code AR9640, Leica Biosystems) for 15 min at 95°, and Protease treatment for 5 min. The following RNAscope 2.5 LS probes (Advanced Cell Diagnostics) were used: rat-specific probes targeting mRNA of fibroblast growth factor 23 (FGF23, NM_130754.1), matrix metallopeptidase 2 (MMP2, NM_031054.2), MMP9 (NM_031055.1), interleukin 6 (IL6, NM_012589.2), tumor necrosis factor (TNF, NM_012675.3) and vascular endothelial growth factor A (VEGFA, NM_031836.2). All probes were incubated at 37° for 120 min. RNAscope® 2.5 LS Assay on Leica BOND RX-BROWN (Advanced Cell Diagnostics) was used as pre-amplification system. Subsequently, the reaction was visualized using 3,3-diaminobenzidine (DAB) as brown chromogen (Bond polymer refine detection, Leica Biosystems, Ref DS9800) for 20 min. Finally, samples were counterstained with haematoxylin for 20 min, dehydrated, and mounted with Pertex (Sakura).

### Immunohistochemistry

IHC evaluations were performed on 2.5 μm sections of FFPE tissue mounted onto glass slides, dried, and baked at 60 °C for 30 min. Bond RX (Leica Biosystems) Immunostainer was used for automated staining. All slides were dewaxed in Bond dewax solution (product code AR9222, Leica Biosystems). Antigen retrieval was performed in Tris–EDTA buffer based (code AR9640, Leica Biosystems) for 30 min at 95° for anti-CD3 (1:400, Thermo Fisher MA190582); and in citrate buffer based (code AR9961, Leica Biosystems) for 30 min at 100° for anti-CD20 (1:200, Abcam, ab194970), anti-CD31 (1:30, Abcam ab28364), HLA-DR (1:400, Thermo Fisher MA532232;), anti-CD163 (1:400, Thermo Fisher PA578961) (Additional file 1: Table S1). All samples were then incubated with horseradish peroxidase-polymer for 15 min and subsequently visualized using 3,3-diaminobenzidine (DAB) as brown chromogen (Bond polymer refine detection, Leica Biosystems, Ref DS9800) for 10 min. Following these procedures, samples were counterstained with haematoxylin for 5 min, dehydrated, mounted on Pertex (Sakura).

### Microscopical analyses

Stained slides were digitized with a digital slide scanner (Pannoramic P1000/Panoramic 250, 3DHistech Ltd, Budapest, Hungary). All analyses were performed using a digital slide viewer (caseViewer, 3DHISTECH, Budapest, Hungary) by two independent observers (JH, JR), blinded to treatment and follow-up time. Qualitative light microscopic analysis was performed according to a previously introduced 4-tier grading system (Additional file 1: Table S2.) The presence of specified stained cell types and soluble factors was graded as 0 = none, 1 = mild, 2 = moderate, 3 = severe (Additional file 1: Figs. S1 and S2). Evaluation was performed for pre-defined ROIs: (1) aneurysm neck, (2) aneurysm dome, (3) thrombus, (4) neointima and (5) adjacent vessel (Additional file 1: Fig. S3). If not all five ROIs were identified on the histological slide, the missing one was not graded for that specific factor and ROI. All specimen with the same follow-up time were combined for ROI-specific analyses. ROIs were consolidated (neck and dome; thrombus and neointima) for time-dependent analyses.

### Statistical analysis

Kruskal–Wallis test compared presence of inflammation cells and markers between the different ROIs, and Mann–Whitney test was used for comparison between two groups. For evaluation of surgical characteristics (normally distributed, parametrical values) Student’s *t*-test was used. Data were analyzed and visualized using GraphPad Prism 8 (Version 8.2.0.435, GraphPad software, San Diego, CA, USA). Values are expressed as mean ± standard deviation (SD) (for parametrical values) or median and interquartile range (for non-parametrical values). A *p*-value < 0.05 was considered statistically significant.

## Results

**Fig. 2:**
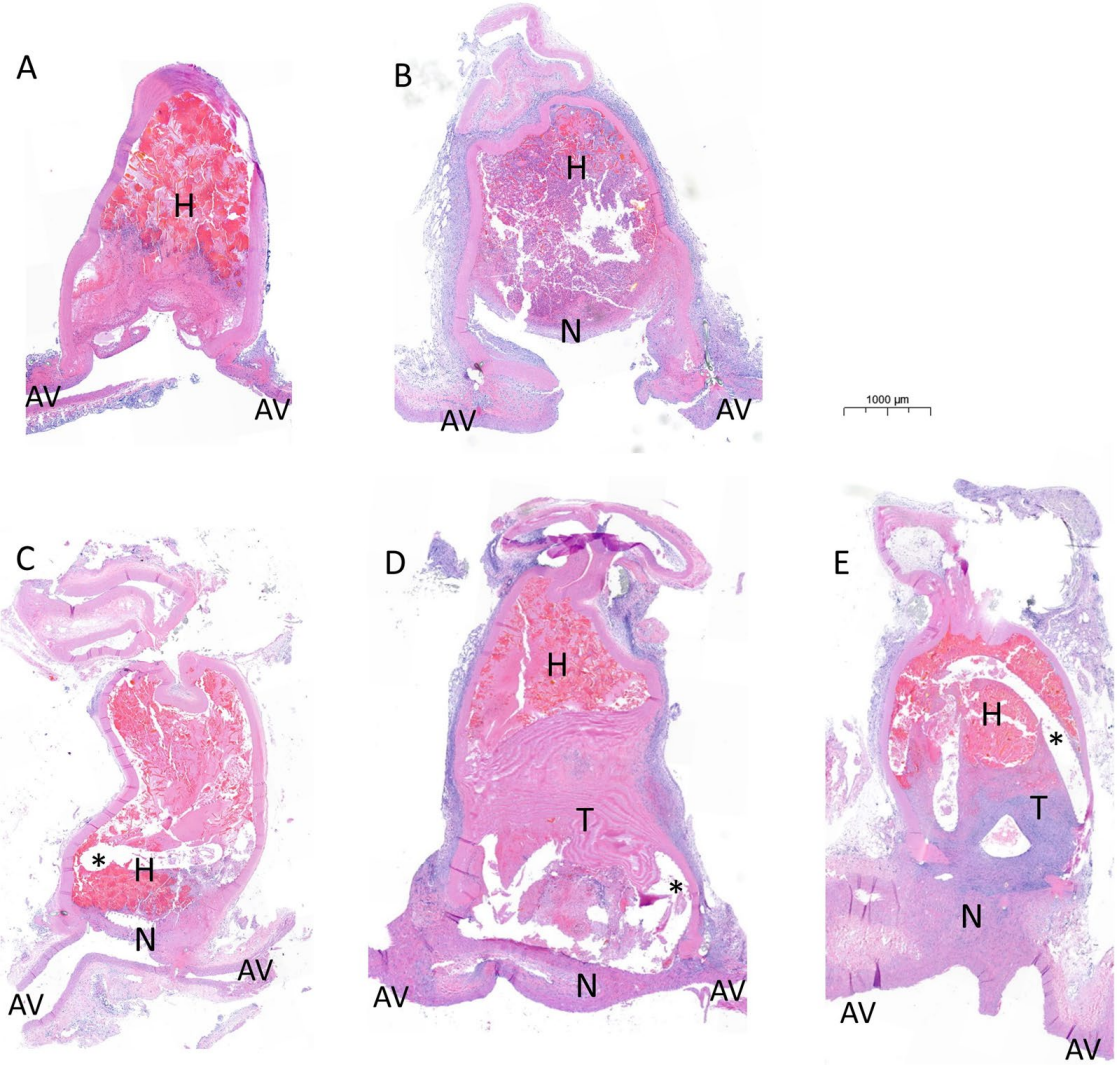
Evolution of aneurysm healing without and with coil treatment. Longitudinal cuts of the aneurysm complex following natural course (top row) and coil (*) treatment (bottom row), 3 days (**A**, **C**), 7 days (**B**, **D**) and 21 days (**E**) into the healing process. Note the absence of cells in the aneurysm wall. After 3 days of coil treatment (**C**) there is already a small tissue layer, separating the lumen of the adjacent vessel (AV) from the early hematoma (H) in the aneurysm sac, which develops into a thick neointima (N). Early hematoma is gradually transformed (**D**) into mature thrombus (T) with only minimal residual hematoma in the top part of the aneurysm sac after 3 weeks (**E**). In the absence of coils, the intra-aneurysmal hematoma shows massive neutrophile invasion, insufficient thrombus maturation and remaining central residual aneurysm perfusion (**B**)

**Fig. 3:**
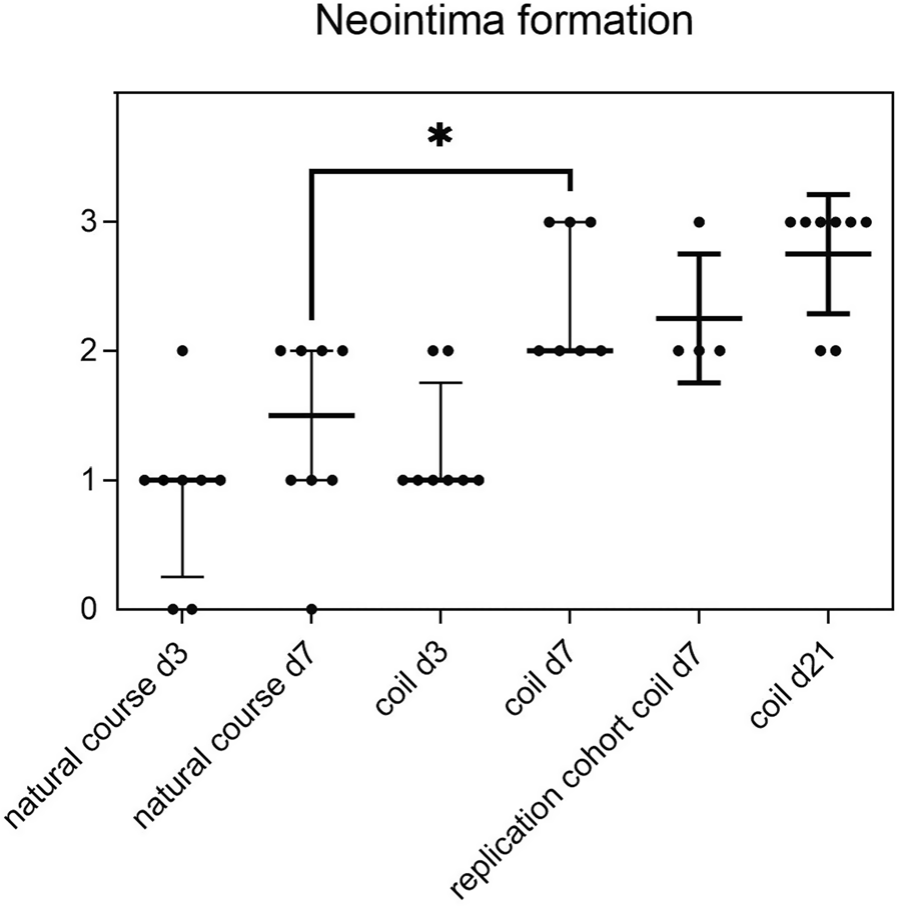
Aneurysm healing reflected by neointima formation as a function of treatment (coiling) and time. No relevant difference between coiled and uncoiled aneurysms was found at three days post-op. However, after seven days those with coil treatment showed significantly stronger neointima formation. Good aneurysm healing reflected by strong neointima formation was confirmed in the replication cohort (FU 7 days) and in the long-term cohort (FU 21 days). *p < 0.05

Overall, coiled aneurysms showed an enhanced pattern of thrombus organization and neointima formation, whereas those without treatment demonstrated a heterogeneous pattern of thrombosis, thrombus recanalization, and aneurysm growth (*p* < 0.001, Fig. 2). Neointima formation was significantly more advanced in coiled aneurysms compared to controls already after seven days (*p* = 0.020, Fig. 3). Good healing status was confirmed in the reproduction and long-term (21-day) cohorts. After 21 days of coil treatment *n* = 7/8 aneurysms had completely healed (macroscopic observation), and only *n* = 1/8 showed a small area of residual perfusion at the neck. There were no relevant differences in operative characteristics between the two treatment arms (Additional file 1: Fig. S4).

### Differences of inflammation between natural course and coiling

Light microscopy showed a pronounced early presence of inflammation cells (Day 3) in untreated aneurysms as compared to coiled aneurysms (Additional file 1: Fig. S5). However, after 7 days of healing, all humoral inflammation markers examined (TNF-α, IL6, MMP-2, MMP-9 and FGF) were markedly increased in coiled aneurysms (Additional file 1: Fig. S6). The distribution of inflammatory cells (i.e., neutrophils) was similar between coil treatment and natural course of healing at Day 7 (*p* = 0.98).

### Evolution of humoral inflammatory factors

**Fig. 4:**
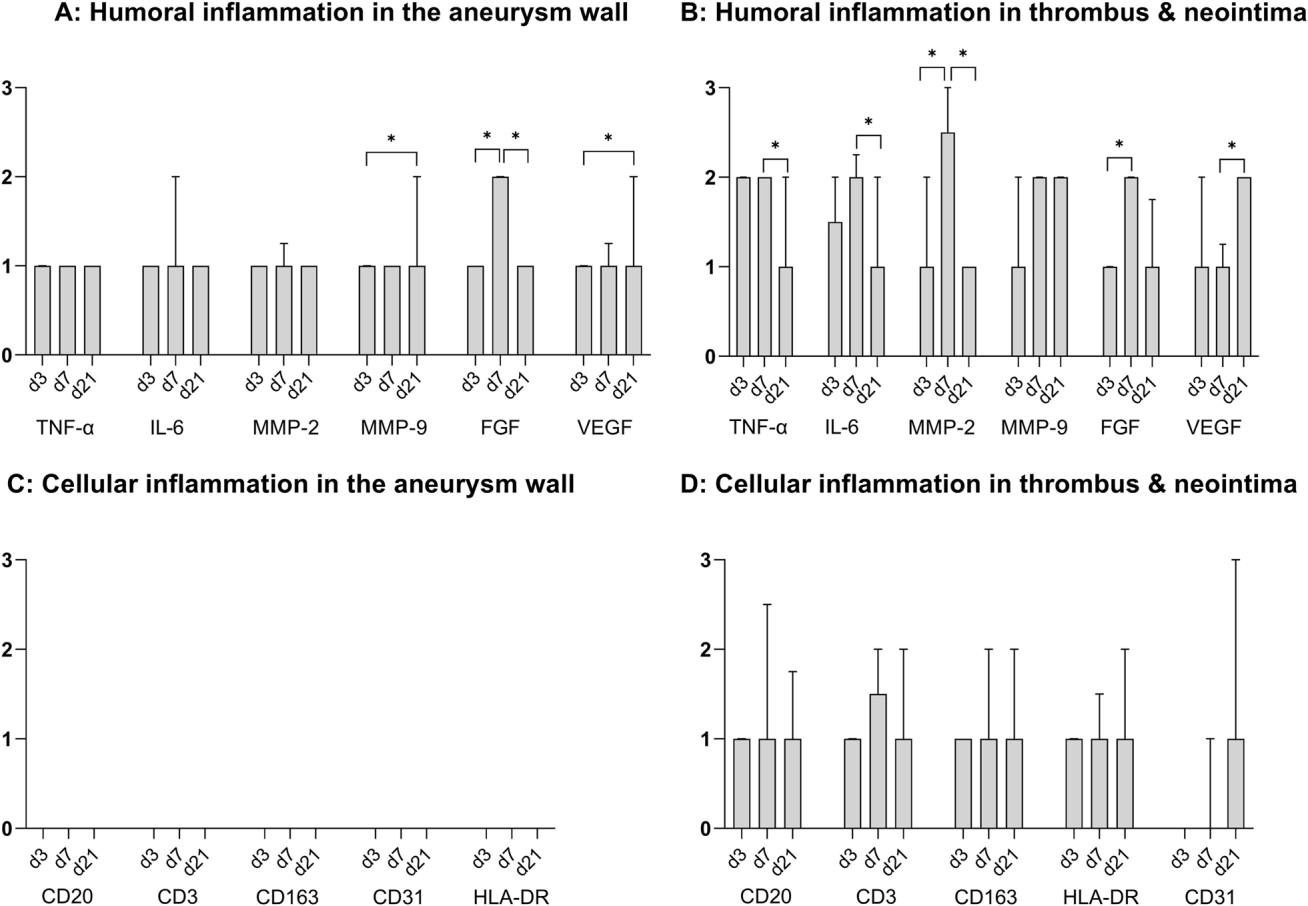
Timely evolution of humoral and cellular inflammation and aneurysm healing after coil treatment. The bar diagrams show median and interquartile range for humoral (panels **A** and **B**) and cellular (panels C and D) inflammation. Cross-sectional data from different animals over time suggest that humoral inflammation peaks at Day 7 for most factors—predominantly in the thrombus and neointima, and to a lesser extend in the aneurysm wall (panels **A** and **B**). On a cellular level (panels **C** and **D**), inflammation cells accumulate in the thrombus, but not in the aneurysm wall. Delayed (after Day 7) increase of VEGF corresponds with increasing endothelialization (CD 31) and thus complete healing over time (*p* = 0.01). *p < 0.05

In coiled aneurysms, humoral inflammation markers gradually increased and peaked on Day 7 (Fig. 4). They were detected ubiquitously in all ROIs, however concentrations were significantly higher in the aneurysm sac and the early neointima than in the aneurysm wall (*p* < 0.0001 for TNF-α, MMP2, and MMP9; *p* = 0.001 for IL6). There were no differences between the neck and dome of the aneurysm wall (Table 1). VEGF concentrations remained low during the first seven days in all ROIs but rose afterwards—predominantly in the thrombus and aneurysm wall.

**Table 1:** Topography of humoral markers

	FGF	Il6	MMP2	MMP9	TNF-α	VEGF
(Unaffected) Adjacent vessel	1 [0.70–1.30]	1 [0.35–1.468]	1 [0.40–1.60]	1 [0.29–1.16]	1 [0.44–1.38]	1 [0.73–1.45]
Aneurysm neck	2 [0.99–1.91]	1 [0.84–1.71]	1 [0.73–1.45]	1 [0.41–1.22]	1 [0.58–1.43]	1 [0.78–1.59]
Aneurysm dome	1 [0.59–1.77]	1 [0.29–1.16]	1 [0.70–1.30]	1 [0.55–1.27]	1 [0.30–0.97]	1 [0.30–0.98]
Thrombus	1 [0.74–1.80]	2 [1.29–2.16]	2 [1.20–2.26]	1 [0.96–1.59]	2 [1.41–2.04]	1 [0.62–1.56]
Neointima	2 [0.99–2.07]	2 [1.44–2.38]	3 [2.30–2.98]	2 [2.00–2.00]	2 [1.41–2.04]	1 [0.73–1.45]
*p*-value (Kruskal–Wallis)	0.3	**0.001**	**< 0.0001**	**< 0.0001**	**< 0.0001**	0.2

Humoral inflammation factors were present in all ROIs, but more pronounced in the thrombus and in the neointima. The table shows median value and lower and upper 95%-confidence interval for Day 7 of coiled aneurysms (0 = none, 1 = mild, 2 = moderate, 3 = severe)

Bold emphasis refelects statistical significance

### Evolution of inflammation cells

Inflammation cells tended to accumulate inside the thrombus and in the neointima, but less in the aneurysm wall (neither in the neck, nor in the dome), or adjacent vessel wall (*p* < 0.001) (Table 2). Concentration of inflammatory cells rose until Day 7 and remained high (Fig. 4, Panel C/D). Endothelial cells accumulated directly in the neointima at a later point (after Day 7) (*p* < 0.0001). Their presence was associated with complete aneurysm healing in coiled aneurysms.

**Table 2:** Topography of inflammation cells

	CD20	CD163	CD3	HLA-DR	CD31
(Unaffected) Adjacent vessel	0 [0.00–0.00]	0 [0.00–0.00]	0 [0.00–0.00]	0 [0.00–0.0.00]	1 [0.22–1.18]
Aneurysm neck	0 [− 0.14 to 0.64]	0 [− 0.21 to 0.49]	0 [− 0.21 to 0.49]	0 [0.00–0.00]	0 [0.00–0.00]
Aneurysm dome	0 [0.00–0.00]	0 [0.00–0.00]	0 [− 0.13 to 0.33]	0 [0.00–0.00]	0 [0.00–0.00]
Thrombus	2 [0.80–2.20]	1 [0.66–1.84]	1 [0.47–2.19]	1 [0.86–1.63]	0 [− 0.15 to 0.37]
Neointima	1 [0.45–2.05]	2 [1.51–2.78]	1.5 [− 0.55 to 3.55]	2 [1.27–2.29]	1 [0.62–2.38]
*p*-value (Kruskal–Wallis)	**< 0.0001**	**< 0.0001**	**< 0.0003**	**<** **0.0001**	**< 0.0001**

Inflammation cells accumulated in the thrombus and in the neointima but not so in the adjacent vessel and in the aneurysm wall. The table shows median value and lower and upper 95% confidence interval for Day 7 of coiled aneurysms (0 = none, 1 = mild, 2 = moderate, 3 = severe)

Bold emphasis refelects statistical significance

### Topographic patterns of factors and cells in a healing aneurysm after endovascular treatment

**Fig. 5:**
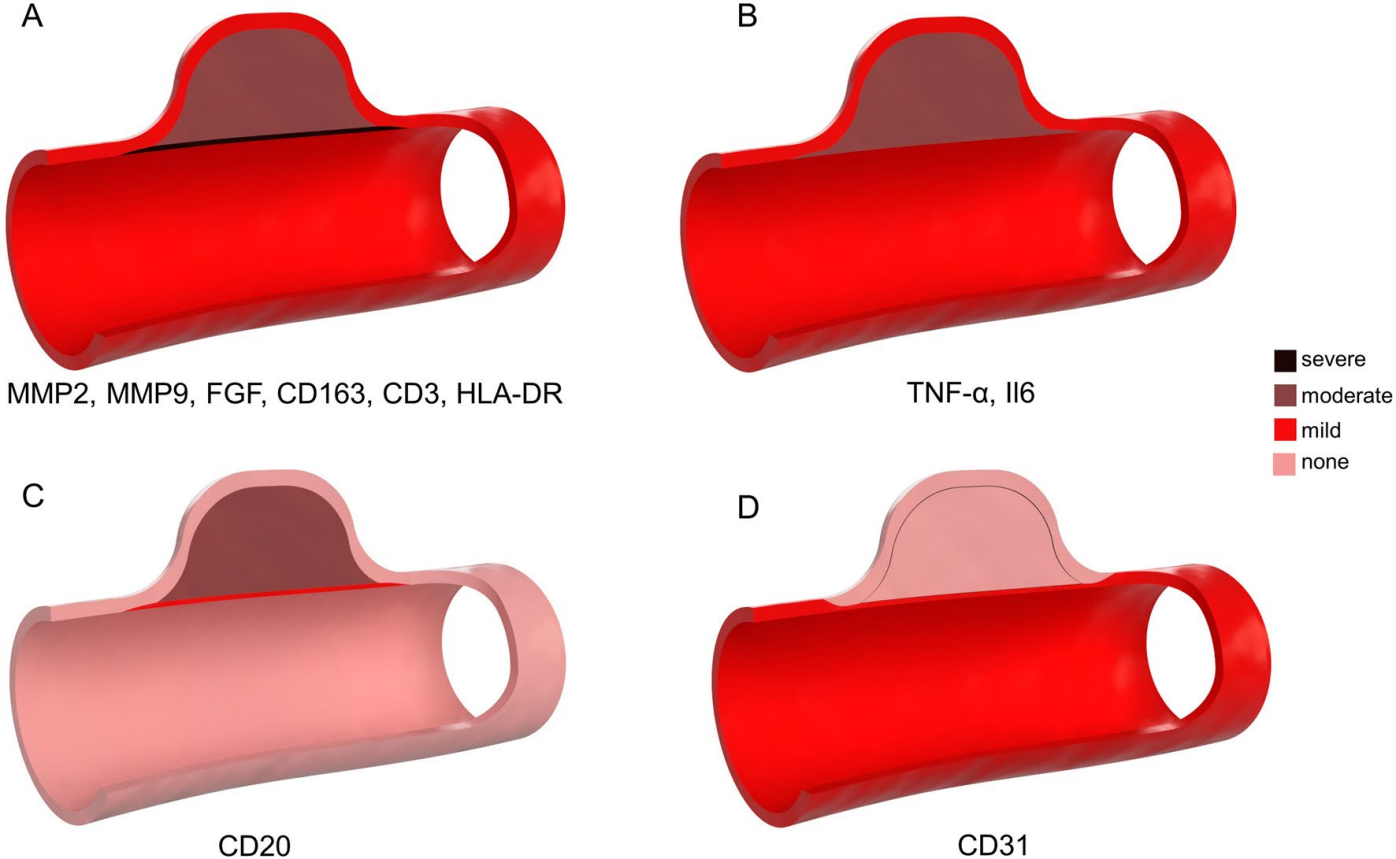
Schematic representation of topographical patterns in a healing aneurysm. Overall, most factors (i.e., MMP2, MMP9, FGF) and cell types (CD163, CD3, HLA-DR) showed the highest accumulation in the neointima followed by the thrombus compartment but were neglectable in the aneurysm wall and in the adjacent vessel (**A**). Few factors (TNF-α, Il6) were equally distributed among the neointima and the thrombus (**B**). The accumulation of B cells (CD20) was higher in the thrombus than in the neointima and scarcely any B cells were found in the adjacent vessel wall (**C**). Lastly, endothelial cells () accumulate directly in the neointima, and their presence is associated with complete aneurysm healing

The distribution of factors examined for the defined ROIs revealed the four following distinctive patterns (Fig. 5): (1) most factors (MMP2, MMP9, FGF) and cell types (CD3, CD163, HLA-DR) showed an accumulation in the neointima > thrombus > aneurysm wall/adjacent vessel; (2) other factors (TNF-α, IL6) were equally high inside the thrombus and neointima; and lower in the aneurysm wall and adjacent vessel; (3) accumulation of B cells (CD20) was more elevated in the thrombus than in the neointima; (4) endothelial cell marker CD 31 was found equally in the unaffected adjacent (healthy) vessel wall and in the maturing neointima, but not inside the thrombus or aneurysm wall. Detailed distribution of each factor and cell type examined according to treatment, follow-up time and ROI is shown in Additional file 1: Figs. S7–S18.

## Discussion

Locoregional inflammation plays a crucial role in aneurysm healing after EVT. The aneurysm wall is not significantly involved in this biological process. In contrast, humoral inflammation cells and markers peak at Day 7 inside the thrombus—and even more so in the early neointima. VEGF is increasingly released after seven days of healing. At the same time, CD31-expressing endothelial cells form a continuous cell layer from the adjacent vessel along the neointima, which is associated with complete aneurysm healing.

### Mural, intraluminal, and neointima inflammatory processes leading to aneurysm healing

Previous studies have shown that a rarefication of aneurysm wall cells leads to a chronic mural inflammation reaction, which triggers further aneurysm growth and rupture [4, 9]. For instance, studies have found that M1-macrophages and mast cells are markedly overexpressed in the wall of ruptured human cerebral aneurysms when compared to unruptured ones [15]. In addition to M1/M2-macrophage imbalance, mural leukocyte infiltration, phenotypic modulation and loss of smooth muscle cells, endothelial dysfunction, and cell death have also been associated with aneurysm formation and rupture [16]. In endovascularly treated aneurysms, however, the thrombus and the neointima are the predominant sites for the same chemokines and cell types to accumulate. These mediators, however, activate different pathways in this location and promote thrombus organization, smooth muscle cell invasion, the differentiation of myofibroblast into secretory and contractile phenotypes, and formation of new endothelialized neointima [8]. Endothelialization of the aneurysm orifice is crucial for complete aneurysm healing and SDF-1α has been shown to enhance endothelial progenitor cell migration and consequently complete aneurysm healing in a rabbit model [17]. A different study investigating in the histological and molecular healing of experimental aneurysms in swine found leukocyte and macrophage infiltration in the thrombus at Day 3, followed by myofibroblast invasion at Days 7–14. Presence of macrophages appeared to be crucial for thrombus organization [18]. Regarding genetics, Aoki et al. showed that genes related to inflammation, extracellular matrix remodeling and apoptosis were dynamically regulated in the wall of experimental aneurysms in rats and substantial differences existed in the gene expression profiles between the intima and the media of these aneurysms [19]. Furthermore, an investigation of gene expressions between the neck and dome of experimental aneurysms after coil embolization in a rabbit model found that overexpression of genes encoding proteases, adhesion molecules, and chemoattractant (but not structural molecules, such as collagen) are associated with good healing [20]. The dual and opposing actions of inflammation factors—destruction or stimulation depending on the site in the aneurysm complex—was also reported in another study of ruptured and unruptured human aneurysms. The authors found expression of VEGF-receptors in the aneurysm fundus to be associated with mural T cell and macrophage infiltration, as well as enhanced organization of luminal thrombosis [21].

### Differences in inflammation between coil and flow diverter treatment

Regarding different EVT, it is interesting to note that the expression of inflammation factors varies substantially between conventional coil embolization and flow diverter (FD) treatment. Specifically, in experimental rabbit aneurysms, molecules associated with wound healing were found four times more often in coiled aneurysms than following FD treatment [22]. This suggests that aneurysm healing after coiling depends predominantly on intra-aneurysmal thrombus organization, whereas healing after FD depends rather on direct endothelial cell proliferation along the scaffold, of cells originating predominantly from the parent artery (and potentially circulating progenitor cells) [1, 2]. Furthermore, genes related to inflammation (TNF-α, monocyte chemoattractant protein 1) were upregulated, whereas proteinases (MMP 2 and 9) and structural proteins (collagens and fibronectin) were expressed at lower levels in FD treatment compared with coiled aneurysms [23]. FD procedures with concomitant coiling, however, led to decreased levels of active MMP 9, and the authors concluded that intra-aneurysmal thrombus is the site of MMP activation, which is diminished in the presence of coils [6].

### Future therapeutical implications

The healing of a post-coiled aneurysm is a dynamic process. Many different strategies have been suggested to enhance the biological healing phase using pharmaceutical drugs which target the aneurysm wall or provide a systemic mode of action (i.e., aspirin) [24]. However, in light of the above-mentioned findings, the intra-aneurysmal lumen would be a promising location to administer pharmaceutically active substances. For instance, coil coatings with IL-6 or with osteopontin have been shown to positively regulate monocyte chemotactic protein 1, and were associated with good aneurysm healing in a mouse model [25]. Coils carrying growth factors such as FGF, tissue growth factor β (TGF-β), VEGF and others [26, 27], or tissue allografts (fibroblasts, stem cells) [28, 29] have also shown promising results in preclinical experiments, but have yet to be tested in humans. Coating other endovascular devices such as WEB devices, stents, or FD, could increase the potential effects by delivering the pharmaceutical substance more precisely to the site of neointima formation.

### Strengths and limitations

This study revealed a distinct topographic distribution of inflammation factors in addition to a timely evolution in a healing aneurysm after EVT. Results are extremely robust, confirmed by a long-term cohort and internally validated by a replication cohort. However, aneurysm wall remodeling and thrombus formation are not determined exclusively by locoregional inflammation. For instance, flow characteristics influenced by aneurysm size and aneurysm-parent artery configuration may play an important role. This effect was minimized through the use of an aneurysm model with highly standardized aneurysm dimensions. Although a small interindividual variance in dimensions between experimental animals remains, this is unlikely to have had a perceptible influence. Still, the aneurysm model chosen does neither reflect the pathogenesis of human intracranial aneurysms nor all aspects of biological aneurysm healing after EVT. Since the aneurysm wall plays an important role in this process, it was of paramount importance to use a model that allows for highly degenerated, decellularized wall conditions resulting in aneurysm remodeling, growth and ultimately rupture [8, 30, 31]. Furthermore, sex hormones have also been shown to significantly influence the aneurysm healing process [32]. Male rats only were used in this study in order to avoid this potential confounding factor. Finally, as inflammation may be triggered by aneurysm creation surgery, inflammation in the experimental group must be considered relative to the control group (natural course).

## Conclusion

A timely and topographically distinct, well-controlled inflammation reaction plays a crucial role in aneurysm healing after EVT. The aneurysm wall remains unimportant in this healing process. Instead, thrombus organization is hallmarked by an invasion of cells and release of proinflammatory cytokines and even more so formation of the early neointima. Neointima maturation is characterized by luminal endothelialization and thus sealing of the parent vessel from the former aneurysm orifice. Understanding this topographic distribution may pave the way for more specific strategies to enhance aneurysm healing—for instance, by targeting the intra-aneurysmal regenerative inflammatory process with covered coils or enhance cell migration from the adjacent vessel with biologically active FDs.

## Supplementary Information

**Additional file 1.** Supplementary Tables and Figures.

## Data Availability

All data supporting the findings of this study are available within the paper and its Additional file 1.

## Abbreviations

EVT: Endovascular treatment
IA: Intracranial aneurysm
ROI: Region of interest
