# Supplementary material for: Topographic distribution of inflammation factors in a healing aneurysm
Source: J Neuroinflammation. 2023 Aug 2;20:182. doi: 10.1186/s12974-023-02863-1 (PMC10394867; doi:10.1186/s12974-023-02863-1)

**Additional file to the Manuscript**

**Topographic Distribution of Inflammation Factors in a Healing Aneurysm**

Basil E. Grüter^1,2,3^, Gwendoline Boillat^1,3^, Joshua Hägler^1,3^, Jeannine Rey^1,3^, Stefan Wanderer^1,3^, Michael von Gunten^3,4^, Jose Galvan^5^, Rainer Grobholz^6, 7^, Hans-Rudolf Widmer^3^, Luca Remonda^2,3^, Lukas Andereggen^1,3^, and Serge Marbacher^1,3^

^1^ Department of Neurosurgery Kantonsspital Aarau, Aarau, Switzerland.

^2^ Division of Neuroradiology, Department of Radiology, Kantonsspital Aarau, Aarau, Switzerland

^3^ Program for Regenerative Neuroscience, Department for BioMedical Research, University of Bern, Bern, Switzerland

^4^ Institute of Pathology Laenggasse, Ittigen, Switzerland

^5^ Translational Research Unit (TRU) Institute of Pathology University of Bern, Bern, Switzerland

^6^ Institute of Pathology, Kantonsspital Aarau, Aarau, Switzerland

^7^ Medical Faculty, University of Zurich, Zurich, Switzerland

**Supplementary Methods**

**Sample size calculation**

An a priori sample size calculation based on preliminary results from previous studies suggested a relevant difference between groups of 30% and standard deviation of 21%. Power analysis indicated an ideal group size of n=8 (power 80%, α=0.05, two tailed, β=0.2). The replication cohort was in line with 3R principles chosen at n=4, to only confirm the validity of the most important experimental group.

$$k=\frac{n_{2}}{n_{1}}=1$$

$n_{1}$ **=** $\frac{({\delta_{1}^{2}+ \delta_{2}^{2}}/{K) {(z_{1-\alpha/2}+ z_{1-\beta)}}^{2}}}{\Delta^{2}}$

$n_{1}$ = $\frac{({{2.1}^{2}+ {2.1}^{2}}/{1) ({1.96+0.84)}^{2}}}{3^{2}}$

$n_{1}$ = 8

$n_{2}$ = K * $n_{1}=8$

**Additional Tables**

**Table S1: Immunostainings for inflammation cells**

Immunostainings for specified inflammation cells was performed according to the antibodies shown below.

| **Antibody** | **Targeted cell type** |
| --- | --- |
| Anti-CD3 | T-cells |
| Anti-CD20 | B-cells |
| Anti-HLA-DR | M1-macrophages |
| Anti-CD163 | M2-macropahges |
| Anti-CD 31 | Endothelial cells |
| Actin, Smooth Muscle (1A4) Mouse Monoclonal Antibody | Smooth muscle cells, myofibroblasts |
| Monoclonal Mouse Anti-Human Von Willebrand Factor, Clone F8/86 | Endothelial cells |

**Table S2: Histopathological analysis**

# The following histopathological characteristics were assessed on conventional hematoxylin‑eosin (HE), Masson-Goldner trichrome (MASA), and smooth muscle actin (SMA) staining, and scored with a previously published 4-tier system, according to *Marbacher et al. Loss of mural cells leads to wall degeneration, aneurysm growth, and eventual rupture in a rat aneurysm model. Stroke; A Journal of Cerebral Circulation. 2014;45:248-254. doi: 10.1161/STROKEAHA.113.002745*.

|  | **Criterion** | **Numeric** |  | **Criterion** | **Numeric** |
| --- | --- | --- | --- | --- | --- |
|  |  |  |  |  |  |
| **Neointima formation** | | | **Neutrophils in the thrombus** | | |
|  | none | 0 |  | none | 0 |
|  | organizing thrombus | 1 |  | mild | 1 |
|  | organizing thrombus and neointima formation | 2 |  | moderate | 2 |
|  | mature neointima | 3 |  | severe | 3 |
|  |  |  |  |  |  |
| **Aneurysm wall inflammation** | | | **Aneurysm wall cellularity** | | |
|  | none | 0 |  | none | 0 |
|  | few (1-3 spots) | 1 |  | few (1-3 spots) | 1 |
|  | many (> 4 spots) | 2 |  | many (> 4 spots) | 2 |
|  | ubiquitous | 3 |  | ubiquitous | 3 |
|  |  |  |  |  |  |
| **Periadventitial inflammation** | | | **Periadventitial fibrosis** | | |
|  | none | 0 |  | none | 0 |
|  | mild | 1 |  | mild | 1 |
|  | moderate | 2 |  | moderate | 2 |
|  | severe | 3 |  | severe | 3 |

**Additional Figures**

**Figure S1: Soluble factors rating**

Chromatin stained in-situ hybridization of soluble factors (FGF23, MMP2, MMP9, Il6, TNF, VEGFA) were graded as: none (top left), mild (few spots, brown arrow, top right), moderate (> 5 spots, bottom left), or severe (ubiquitously, bottom right).


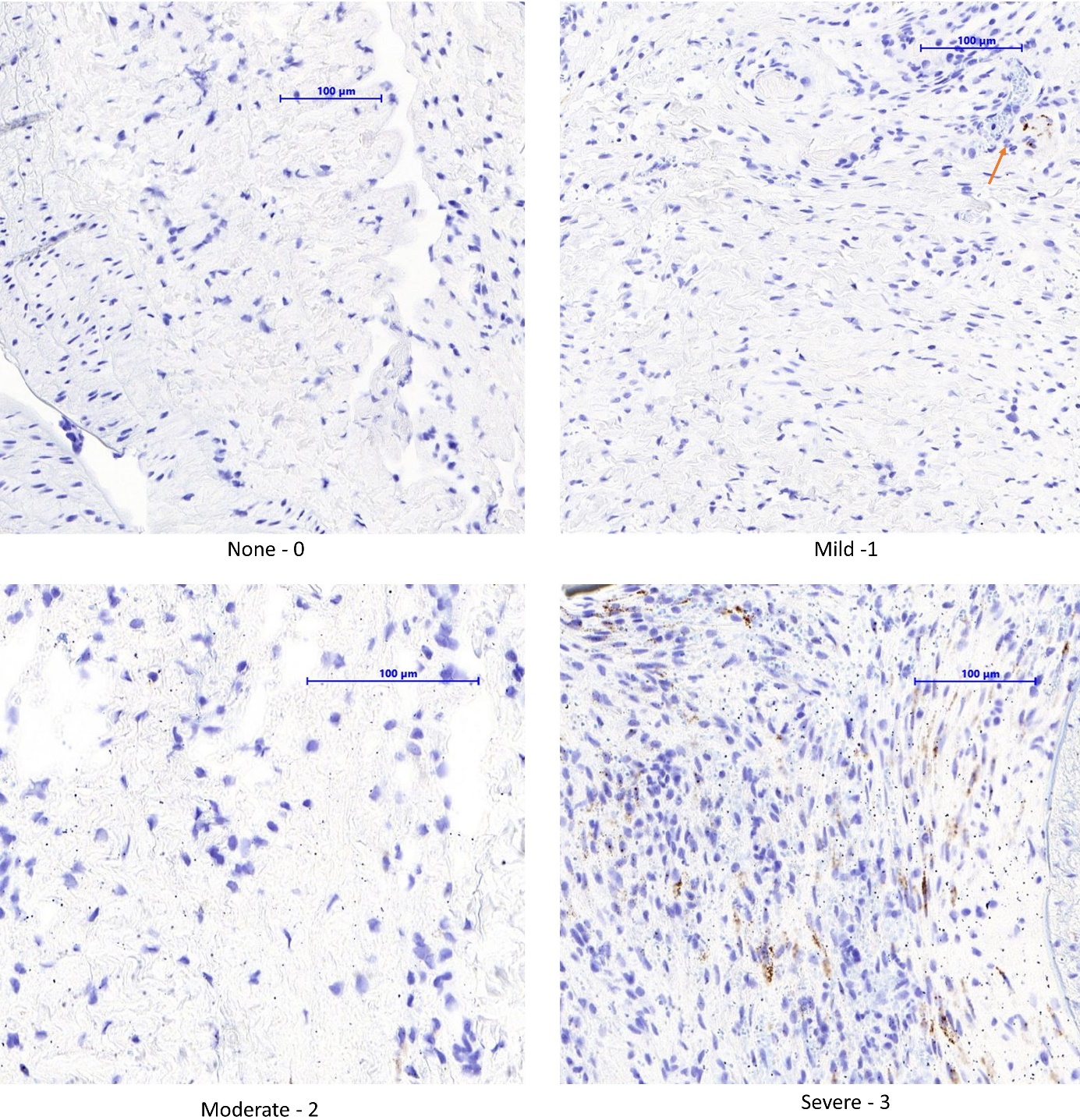


**Figure S2: Inflammation cells rating**

Chromatin stained antigens characterizing immune-cells (CD-20, CD-163, CD-3, HLA-DR, anti-tryptase and CD-31) were graded as: none (top left), mild (few spots), moderate (localized, clustered spots, bottom left), or severe (ubiquitously, bottom right).


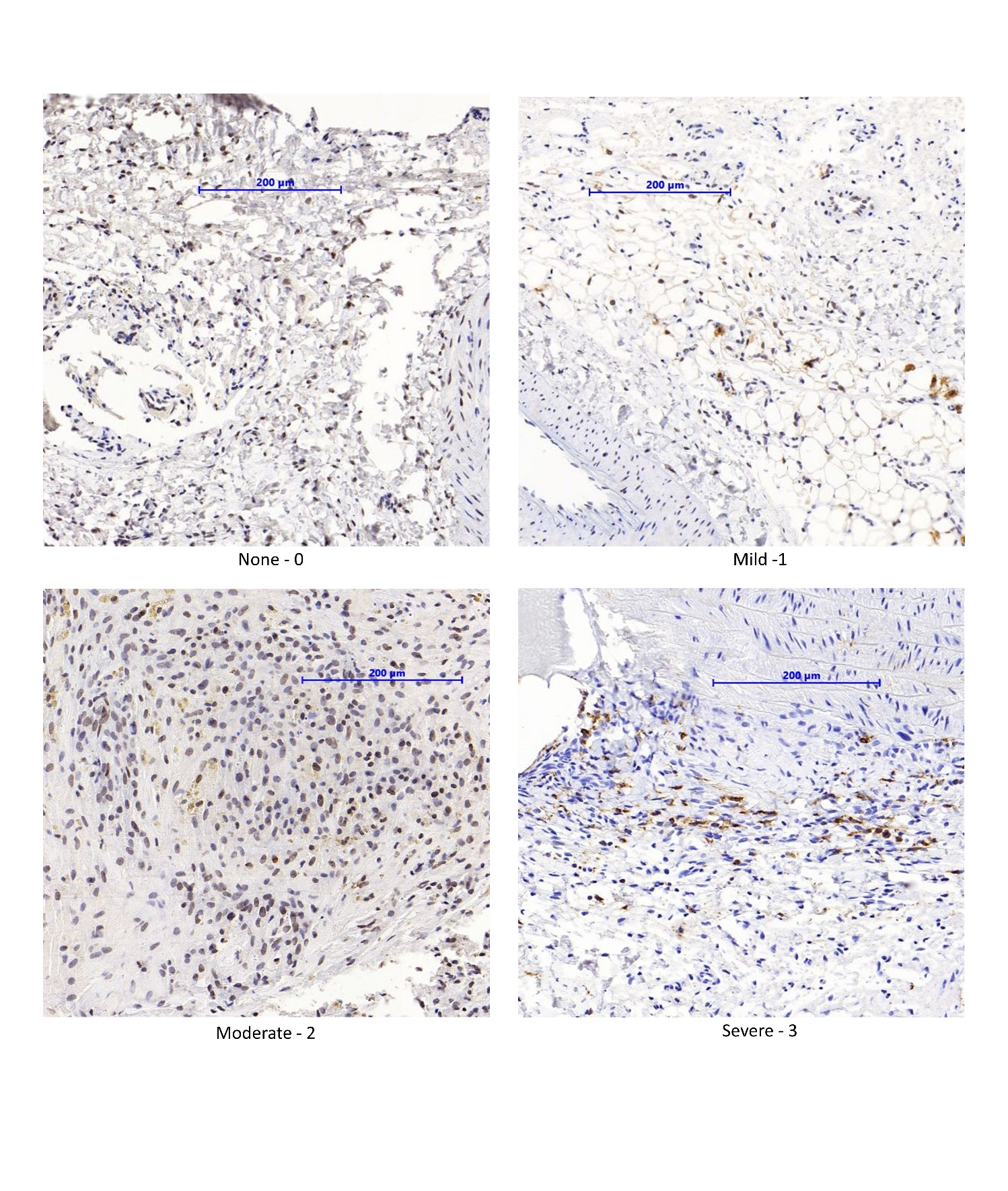


**Figure S3: Regions of interest (ROIs) in the aneurysm complex**

The figure shows a schematic aneurysm with adjacent vessel (red), aneurysm neck (yellow), aneurysm dome (brown), thrombus (green) and neointima (blue).

**Thrombus**

**Aneurysm dome**

**Neointima**

**Adjacent Vessel**

**Aneurysm
neck**


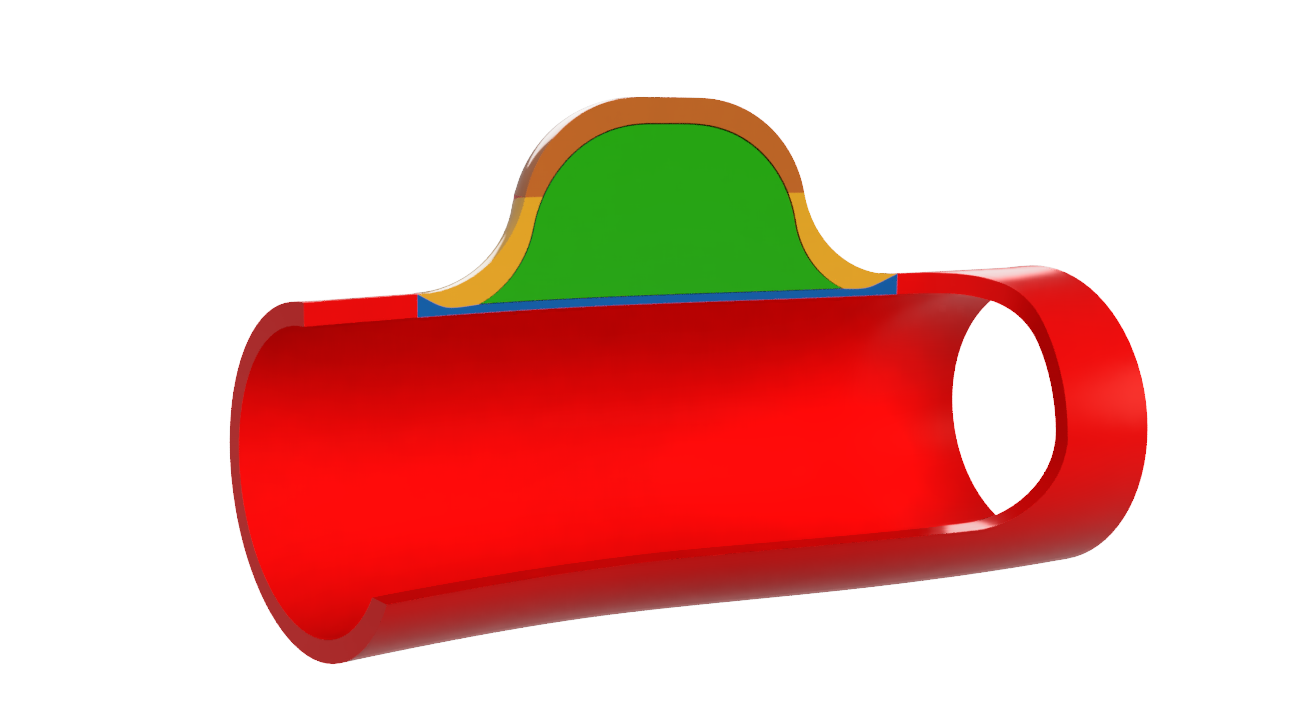


**Figure S4: Operative characteristics**

Although animals allowed to heal naturally were slightly lighter (333 ±13g) than those with coil treatment (345±15g) (p=0.01), there was no significant difference in weight at follow-up. There were no relevant procedural differences between the two groups.


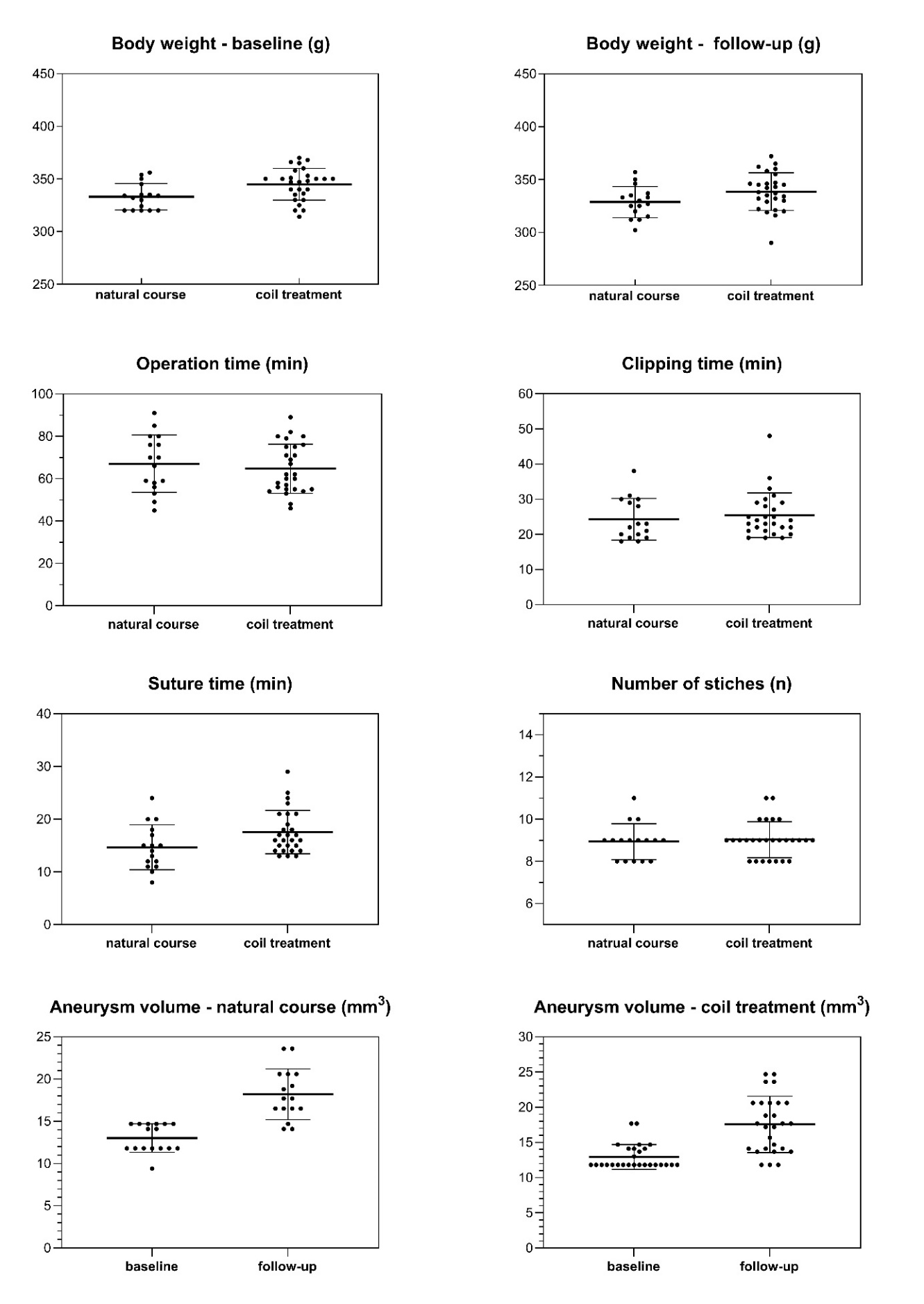


**Figure S5: Light microscopical findings of all experimental groups.**

Untreated aneurysms showed more pronounced inflammation on Day 3 when compared to those with coil treatment. After 7 days, both groups were experiencing inflammation. Neointima formation was significantly stronger in coiled aneurysms as compared to untreated aneurysms at Day 7, which was confirmed in the replication cohort and in the long-term (Day 21) group. * p < 0.05


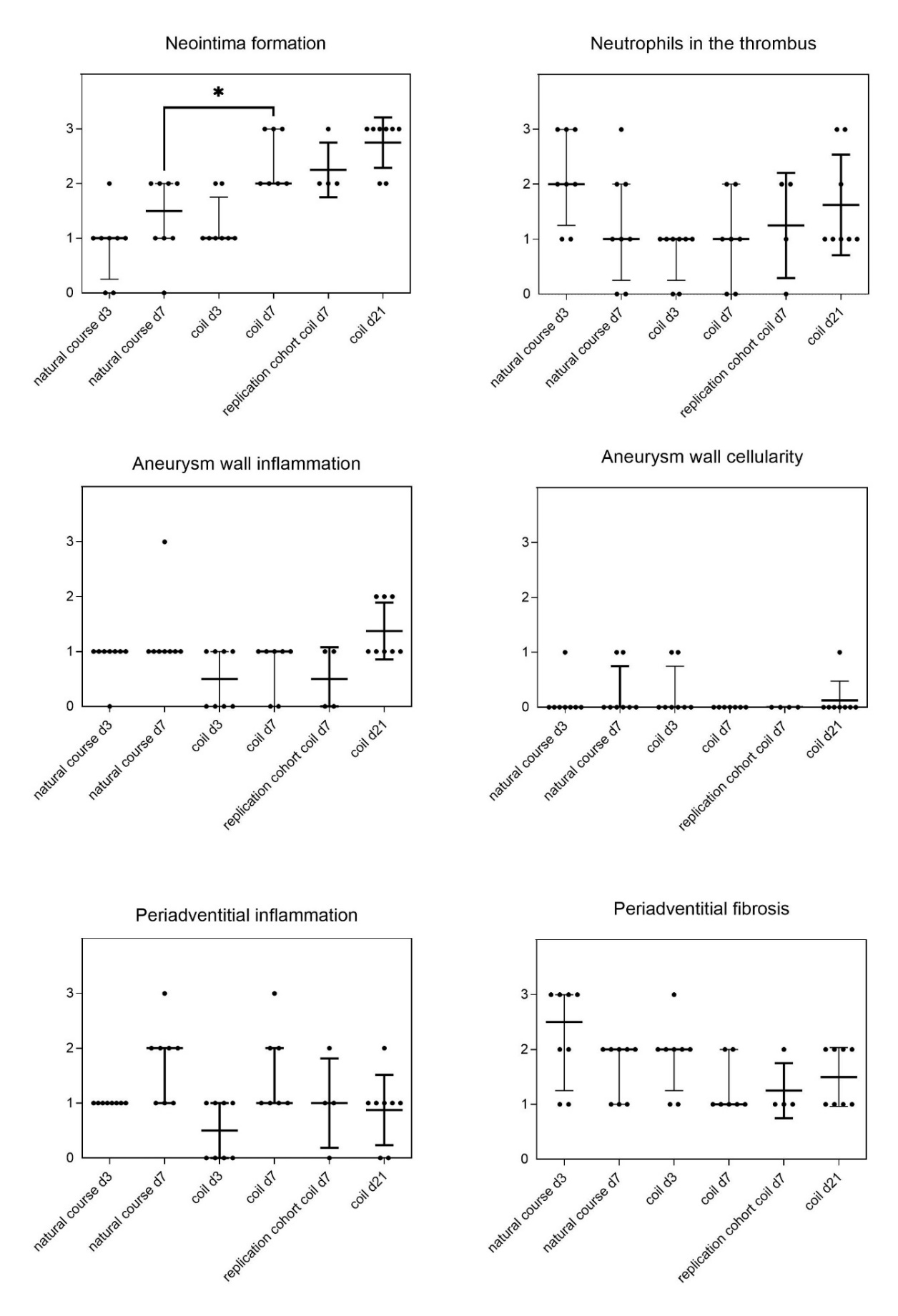


**Figure S6: Humoral inflammation at Day 7 in thrombus and neointima**

The cytokines studied: TNF-α, Il-6, MMP-2, MMP-9, and FGF, were markedly increased in coiled aneurysms when compared to natural course at Day 7.


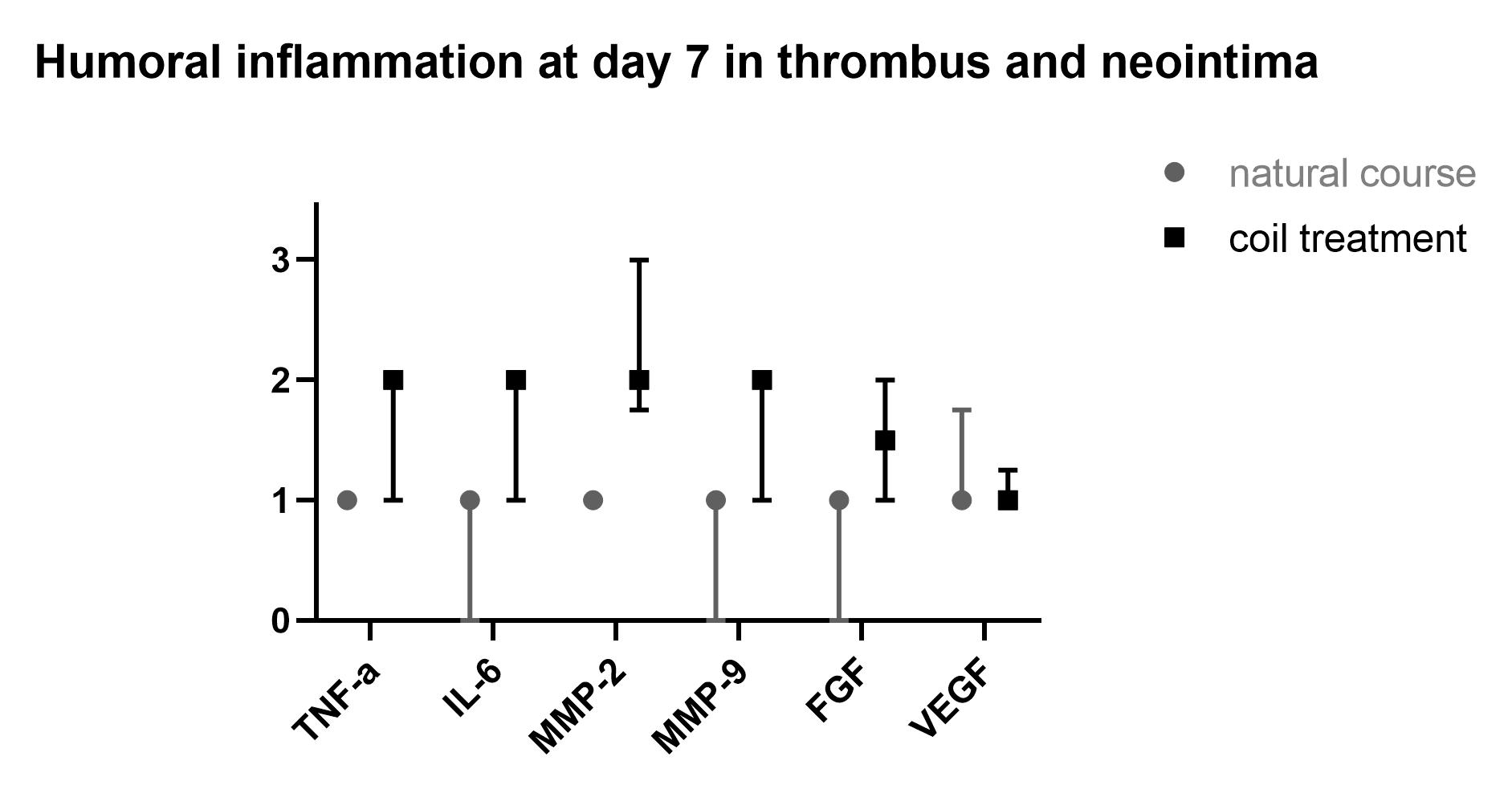


**Figure S7: Distribution of TNF-α**

nc: natural course, coil: coil treatment. Follow-up in days (d)

**
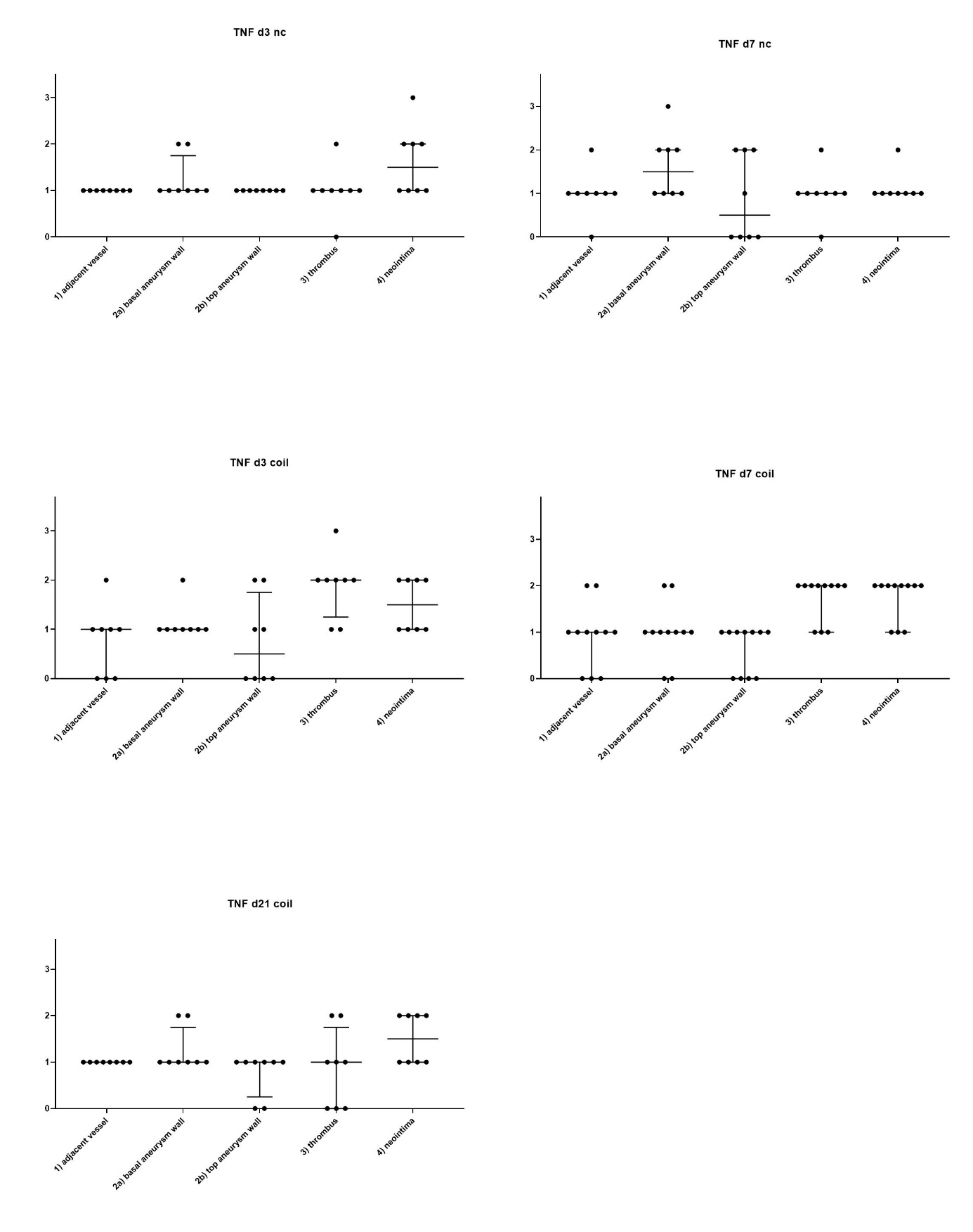
**

**Figure S8: Distribution of Il-6**

nc: natural course, coil: coil treatment. Follow-up in days (d)

**
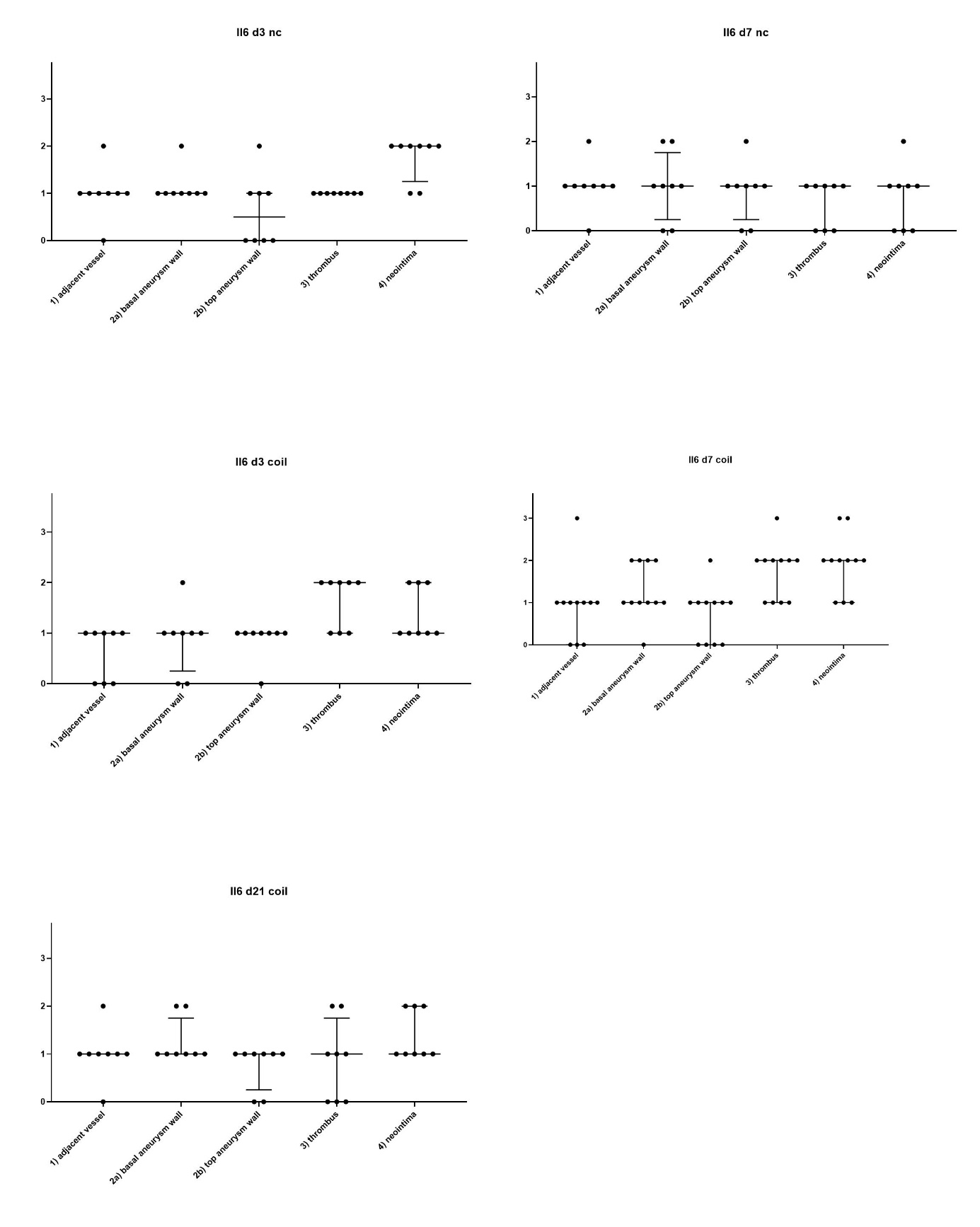
**

**Figure S9: Distribution of MMP-2**

nc: natural course, coil: coil treatment. Follow-up in days (d)

**
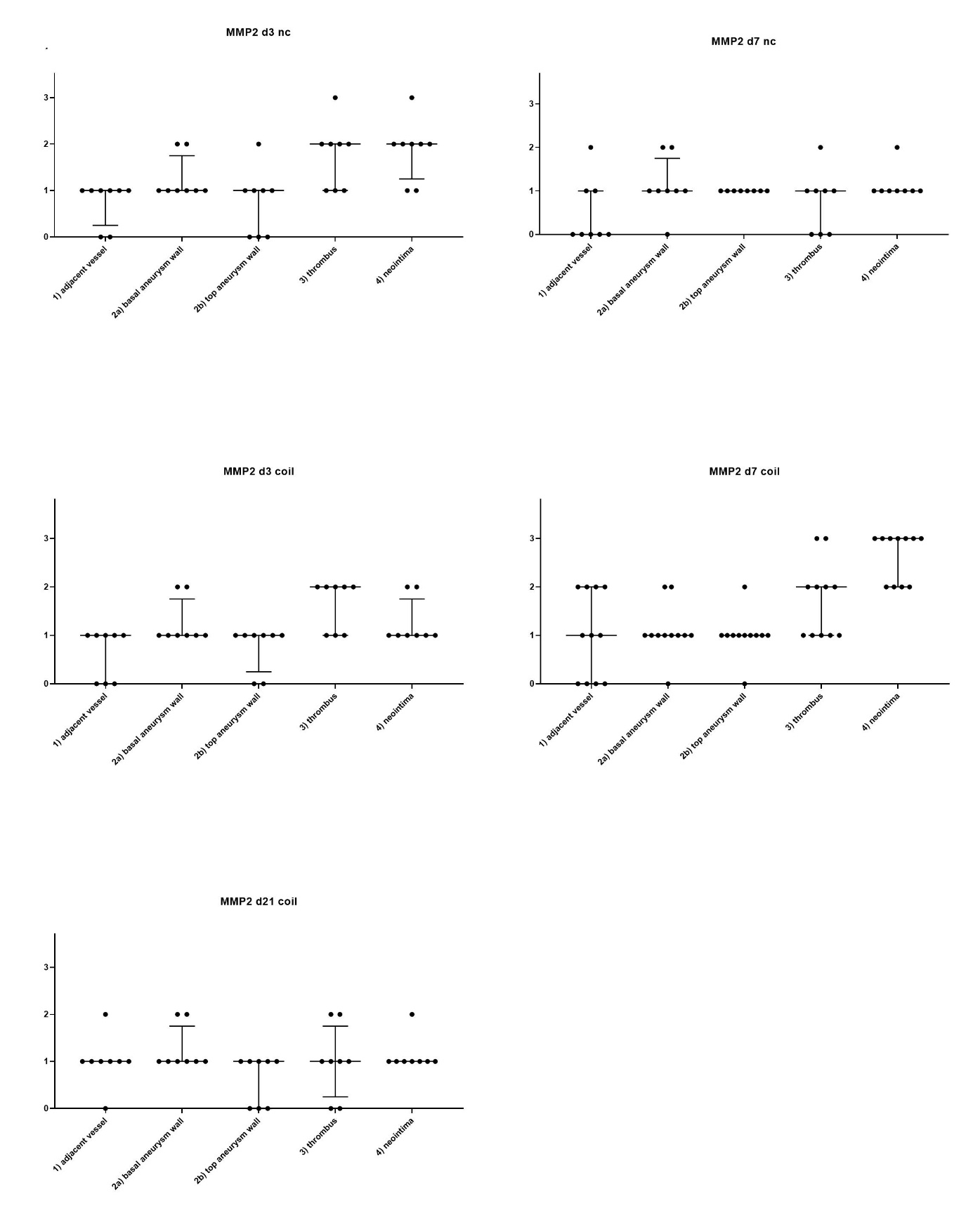
**

**Figure S10: Distribution of MMP-9**

nc: natural course, coil: coil treatment. Follow-up in days (d)

**
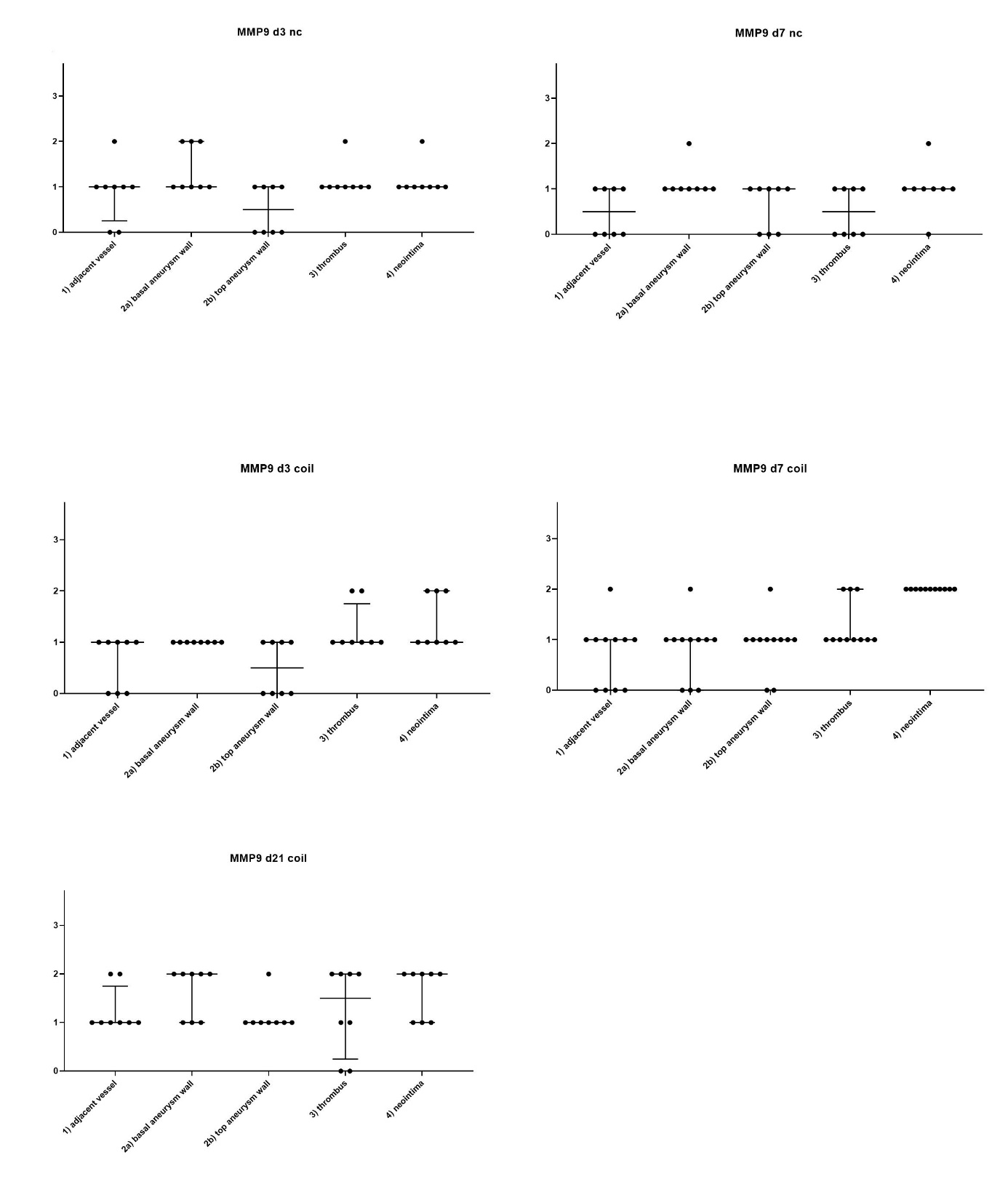
**

**Figure S11: Distribution of FGF**

nc: natural course, coil: coil treatment. Follow-up in days (d)

**
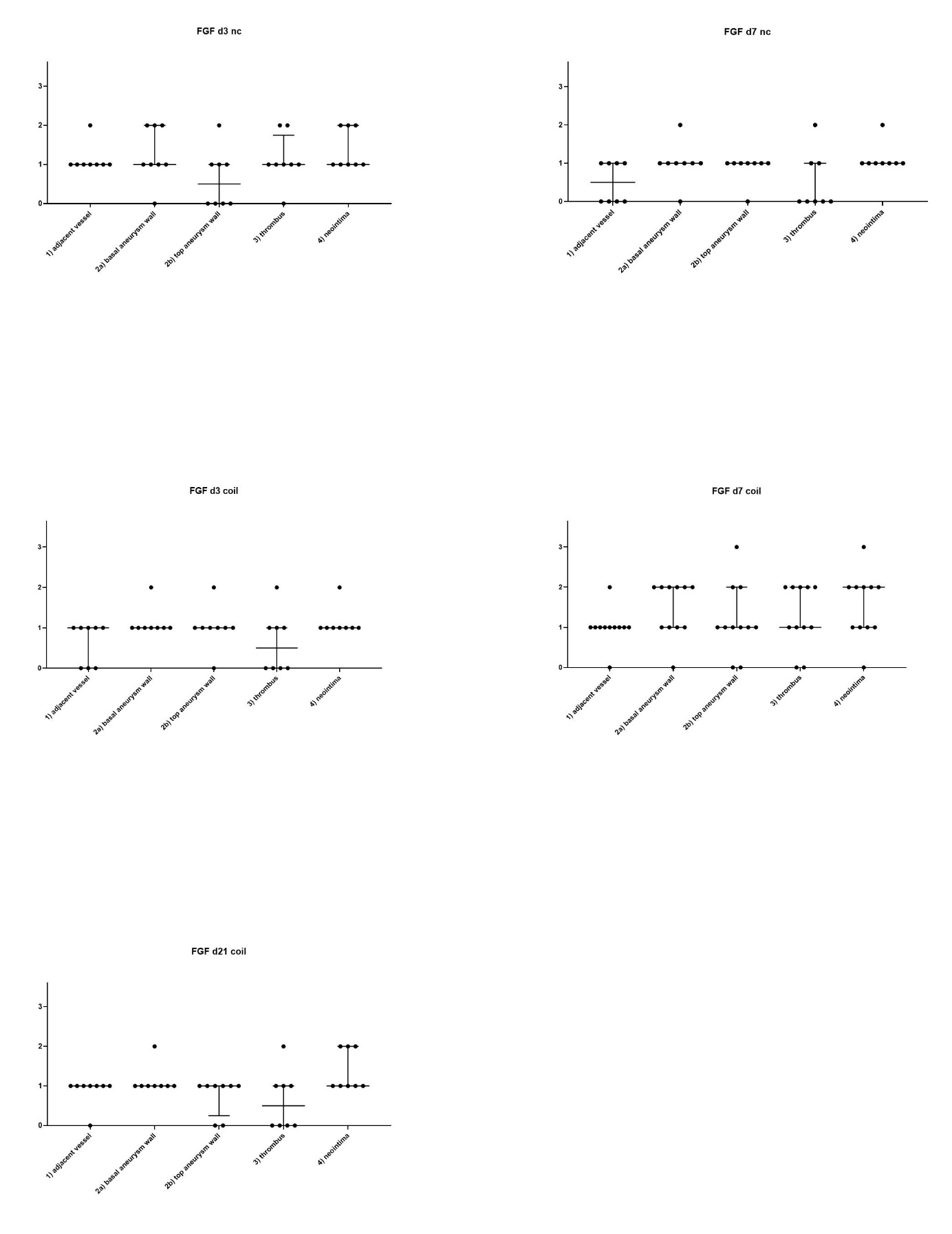
**

**Figure S12: Distribution of VEGF**

nc: natural course, coil: coil treatment. Follow-up in days (d)

**
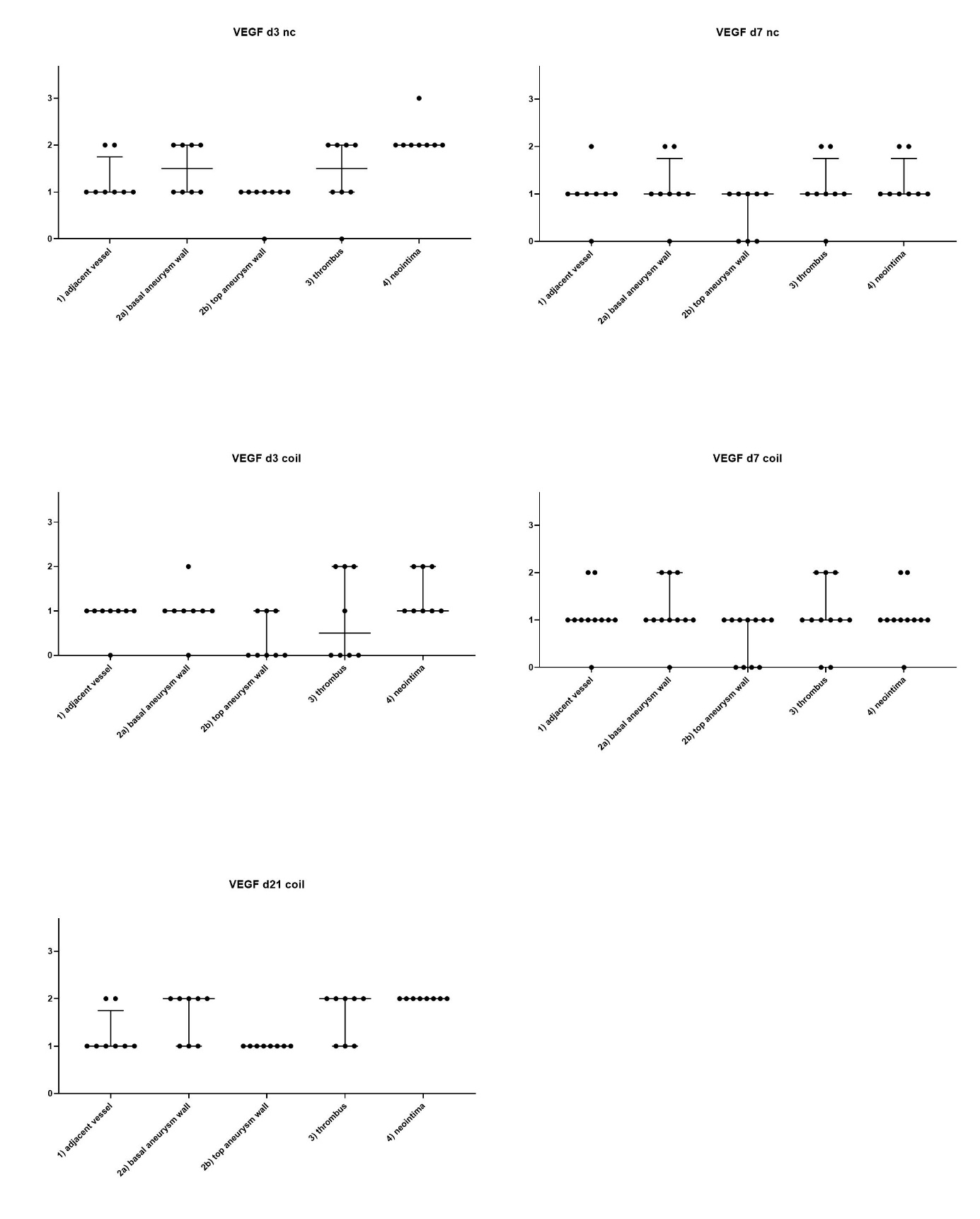
**

**Figure S13: Distribution of CD3+ cells**

nc: natural course, coil: coil treatment. Follow-up in days (d)


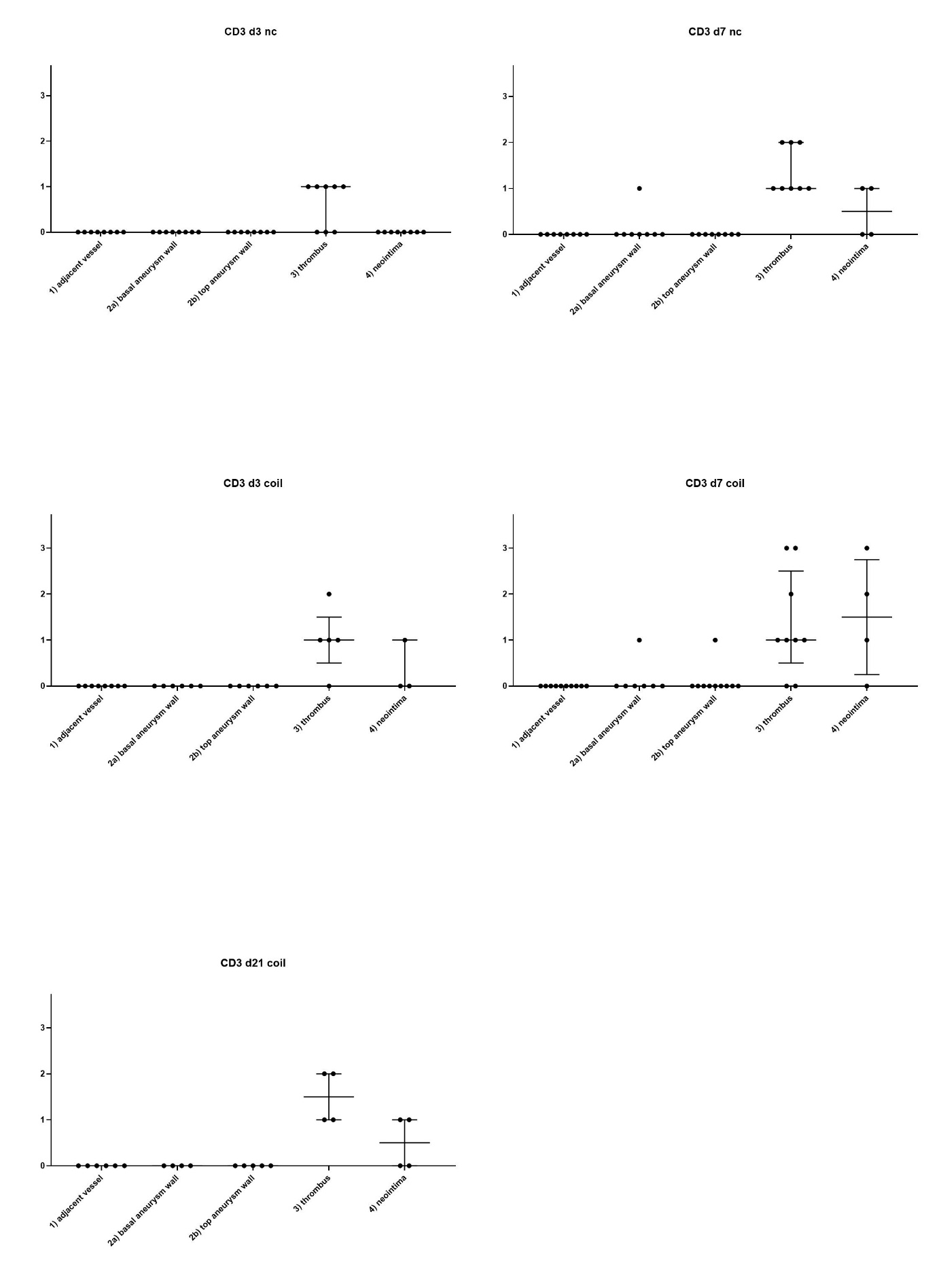


**Figure S14: Distribution of CD20+ cells**

nc: natural course, coil: coil treatment. Follow-up in days (d)


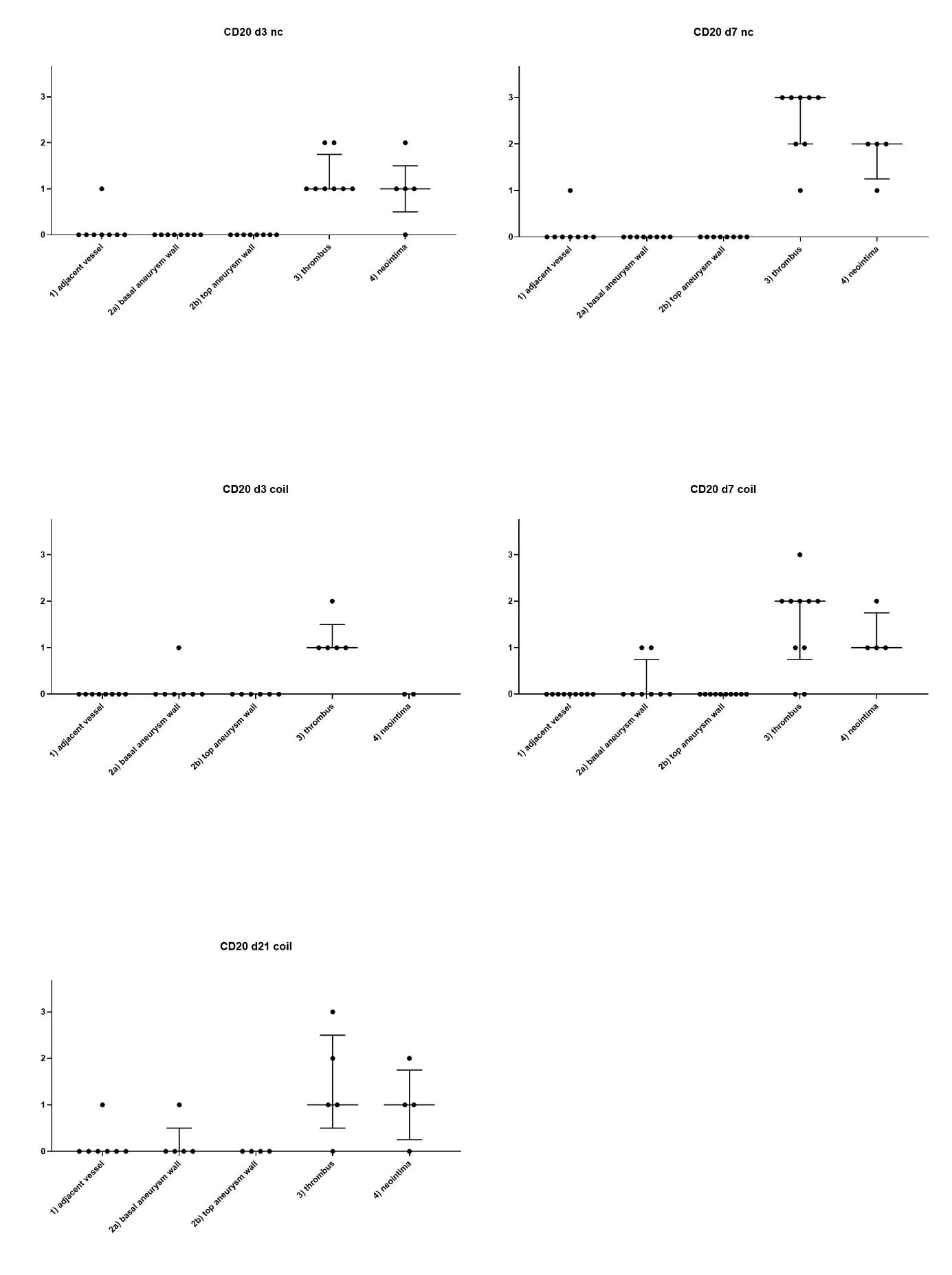


**Figure S15: Distribution of HLA DR**

nc: natural course, coil: coil treatment. Follow-up in days (d)


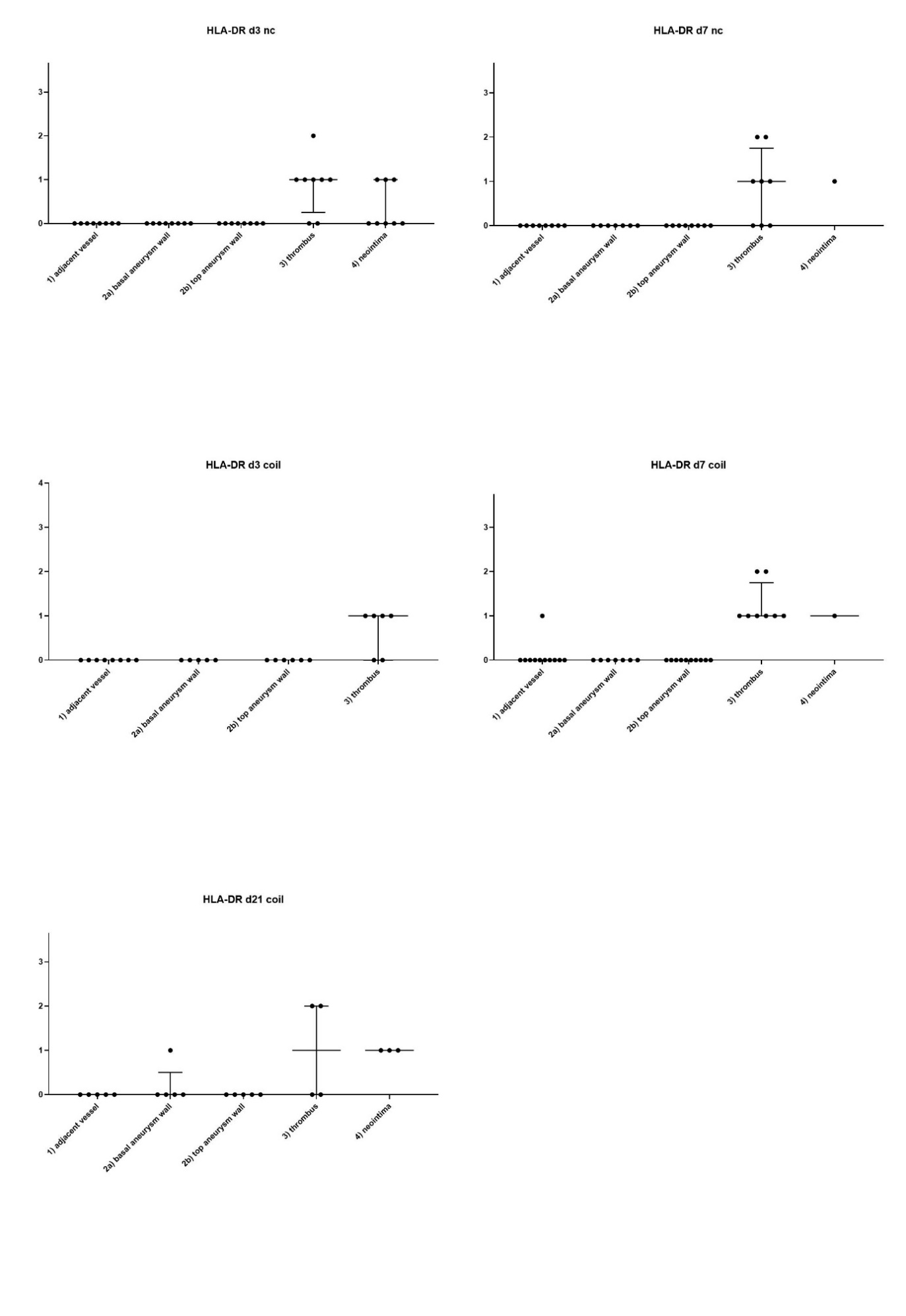


**Figure S16: Distribution of CD 163+ cells**

nc: natural course, coil: coil treatment. Follow-up in days (d)


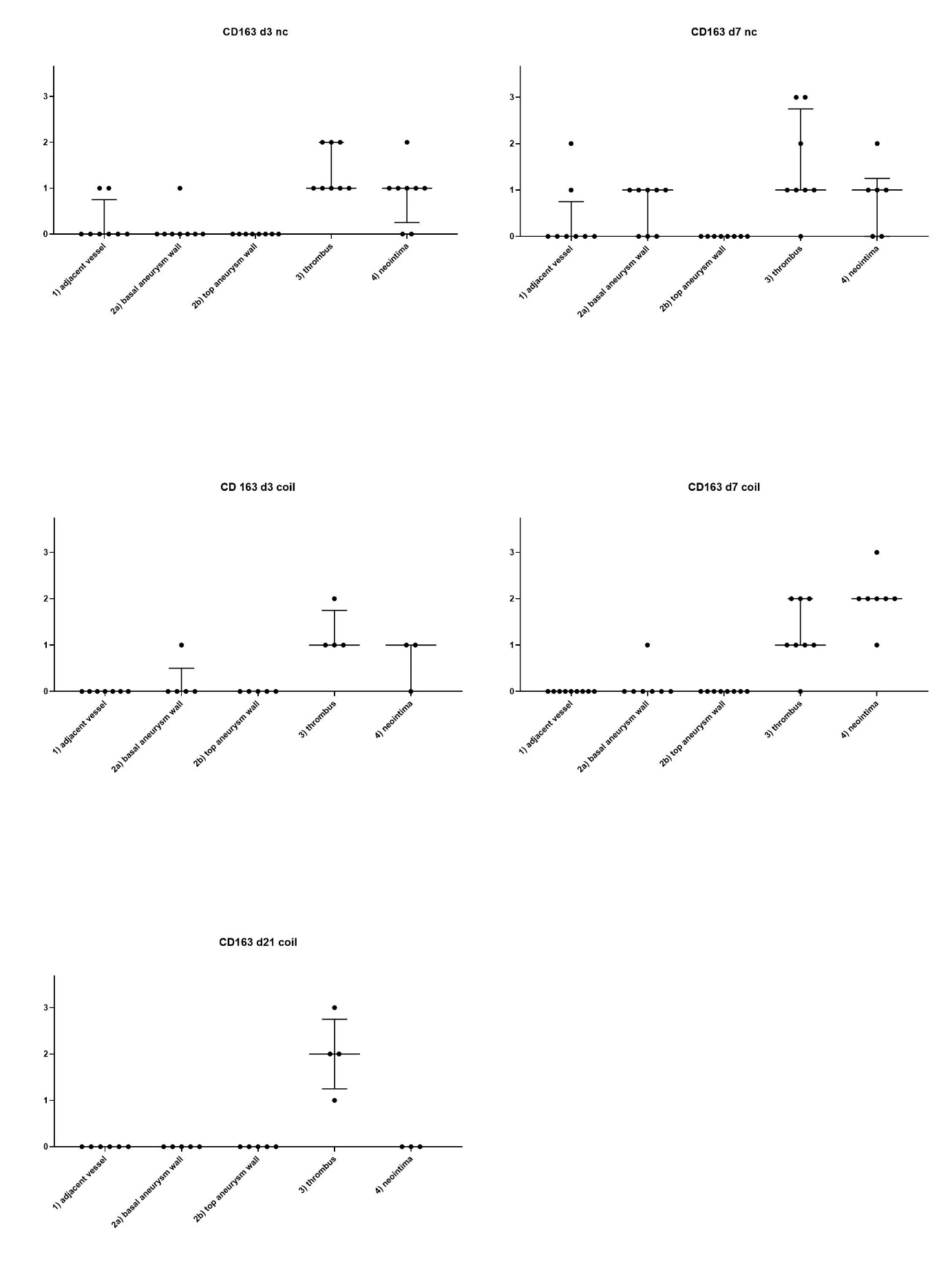


**Figure S17: Distribution of tryptase clone (MAST)**

nc: natural course, coil: coil treatment. Follow-up in days (d)


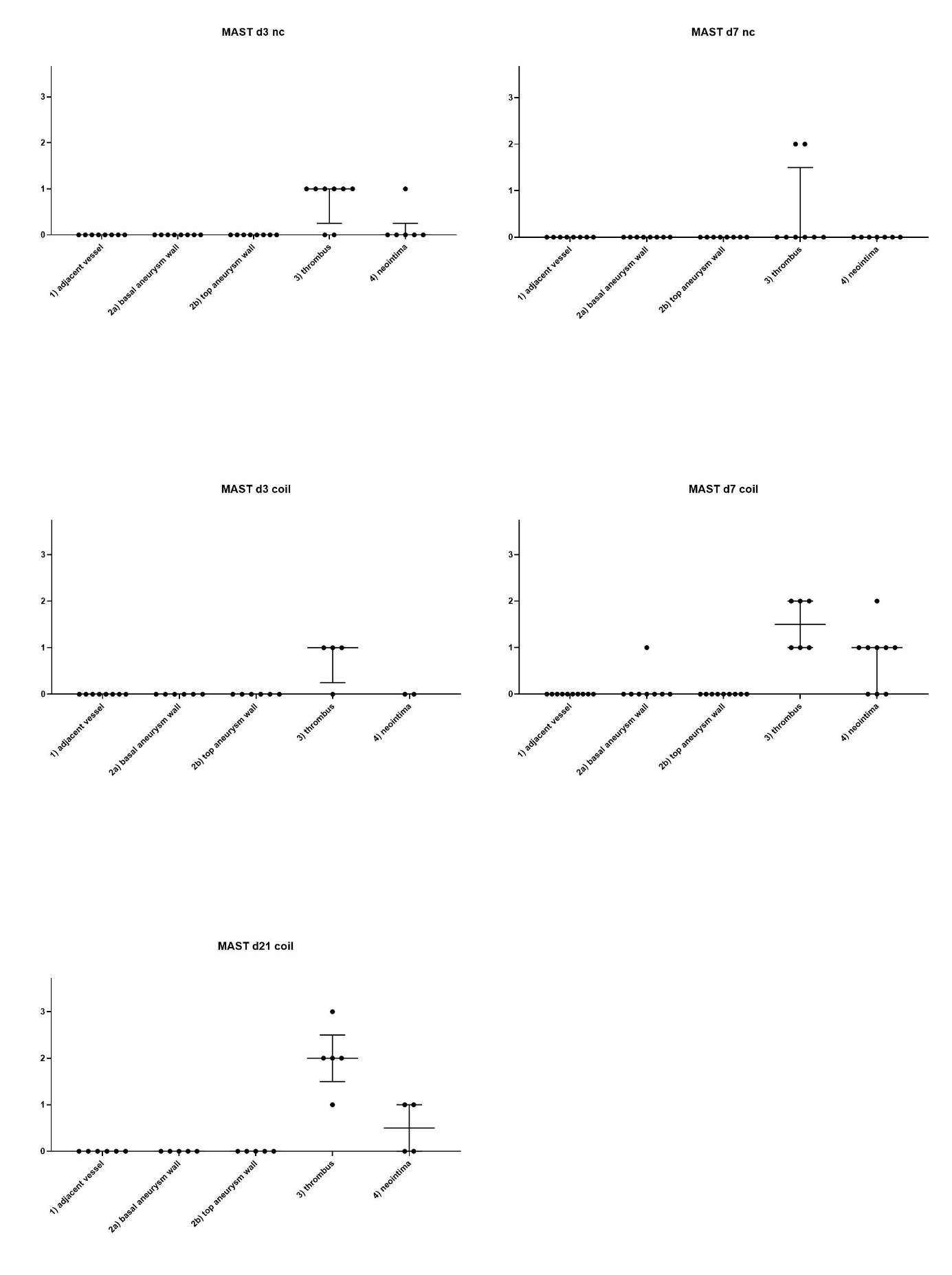


**Figure S18: Distribution of CD31+ cells**

nc: natural course, coil: coil treatment. Follow-up in days (d)


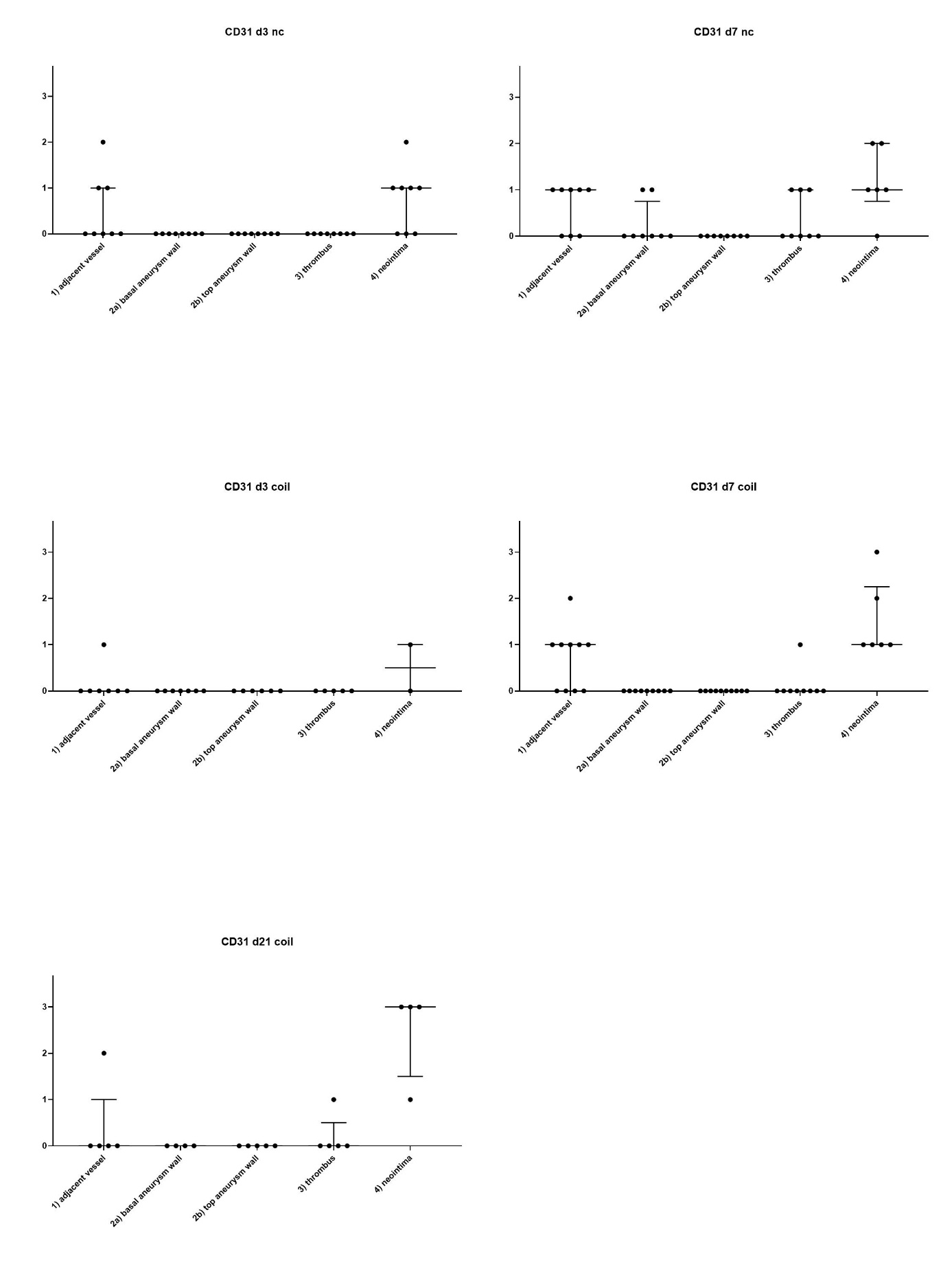

Supplement: Supplementary file 1 — Additional file 1. Supplementary Tables and Figures. [file 12974_2023_2863_MOESM1_ESM.docx]
